# A sensitised RNAi screen reveals a ch-TOG genetic interaction network required for spindle assembly

**DOI:** 10.1038/srep10564

**Published:** 2015-06-03

**Authors:** Alexis R. Barr, Chris Bakal

**Affiliations:** 1 Division of Cancer Biology, The Institute of Cancer Research, 237 Fulham Road, London, SW3 6JB, United Kingdom

## Abstract

How multiple spindle assembly pathways are integrated to drive bipolar spindle assembly is poorly understood. We performed an image-based double RNAi screen to identify genes encoding Microtubule-Associated Proteins (MAPs) that interact with the highly conserved ch-TOG gene to regulate bipolar spindle assembly in human cells. We identified a ch-TOG centred network of genetic interactions which promotes ensures centrosome-mediated microtubule polymerisation, leading to the incorporation of microtubules polymerised by all pathways into a bipolar structure. Our genetic screen also reveals that ch-TOG maintains a dynamic microtubule population, in part, through modulating HSET activity. ch-TOG ensures that spindle assembly is robust to perturbation but sufficiently dynamic such that spindles can explore a diverse shape space in search of structures that can align chromosomes.

To enable faithful chromosome transmission during mitosis, it is essential that cells assemble a robust bipolar spindle with sister chromatids bioriented between two centrosomes. In somatic cells, there are at least four spindle assembly mechanisms that nucleate, polymerise and organise microtubules (MTs). These are the centrosome-, chromatin-, intra-spindle- and acentrosomal MT-organising centre (aMTOC)-mediated spindle assembly pathways. The contribution of each of these pathways to spindle formation at any one time is unclear. When present, centrosomes act as the dominant MT nucleators^1^,^2^. However, even in unperturbed cells, the chromatin-mediated spindle assembly pathway contributes to bipolar spindle assembly^3^, and the MT nucleating protein, γ-tubulin, localises along spindle MTs, implying that the intra-spindle pathway is also active in cells with functional centrosome- and chromatin-mediated spindle assembly pathways^4^,^5^. The existence of at least four partially redundant spindle assembly mechanisms suggests that these pathways have evolved to ensure that MT polymerisation during spindle assembly is robust to perturbation. In support of this model, recent work in *Drosophila* embryos shows that if the activity of the centrosomal pathway is reduced, the contribution of the chromatin pathway to spindle morphogenesis is increased^6^. Whilst the robustness of spindle assembly to perturbation may increase the fidelity of chromosome alignment in fluctuating environments, such robustness must be balanced with the need for spindle MTs to retain their dynamic instability that is crucial for spindles to search a set of structures towards being organised into a bipolar array capable of aligning chromosomes.

MT-associated proteins (MAPs) play key roles in spindle assembly in all eukaryotes as they coordinate the activation of different spindle assembly mechanisms by directly coupling MT dynamics to spatial and temporal cues^7^. MAPs can promote MT assembly/disassembly, MT stabilisation/destabilisation, MT bundling, as well as acting as motors to move MTs past each other, and as molecular scaffolds between MTs and other subcellular structures^8^,^9^,^10^. ch-TOG/CKAP5 belongs to the highly conserved XMAP215 family of centrosomal and MT-binding proteins and is required for bipolar spindle formation in human cells^11^,^12^,^13^,^14^,^15^. XMAP215 family members are also involved in spindle assembly in Fission yeast (Alp14;^16^), Budding Yeast (Stu2p;^17^), *Drosophila* (Minispindles;^18^), and *C. elegans* (Zyg-9;^19^,^20^,^21^). XMAP215 family members promote the formation of long MTs, and can act as both MT stabilisers and destabilisers, suggesting that they act as important anti-pause factors to maintain overall MT dynamics^14^,^22^,^23^,^24^,^25^,^26^,^27^,^28^. ch-TOG is involved in several different aspects of spindle assembly in human cells, as it promotes centrosomal MT growth, maintains centrosomal MT dynamics and regulates kinetochore fibre tension^29^,^30^,^31^. The different effects of ch-TOG on spindle assembly are likely, in large part, to be dictated through complex interactions with other MAPs that occur in ways that are highly regulated in time and space. For example, ch-TOG regulates kinetochore fibre tension by protecting kinetochore MTs from depolymerisation by MCAK^29^,^30^. Moreover, recent experiments have shown that ch-TOG/XMAP215 and EB1 cooperate at MT plus ends to promote MT polymerisation^28^,^32^. However, much remains to be understood regarding how spindle assembly is an emergent property of the actions of multiple independent MAPs.

Whilst single RNAi screens have been used to identify genes that contribute to mitotic spindle assembly^33^,^34^,^35^,^36^,^37^,^38^,^39^,^40^, many single RNAi screens likely have high false negative rates due to factors such as the inherent functional redundancy of biochemical networks^41^. More importantly, through single RNAi screens it is difficult to determine how different genes interact as part of complex networks. As a way to describe networks underpinning diverse cellular behaviours, genetic interaction screens, where two genes are depleted simultaneously, have proven extremely powerful^42^,^43^,^44^,^45^,^46^,^47^,^48^,^49^. Genetic interactions have been particularly successfully implemented in yeast systems where they have been used to characterise interactions between MAPs required for spindle assembly^16^,^47^,^50^. However, similar scalable approaches to understanding spindle assembly in human cells are currently lacking.

To understand how spindle assembly pathways and MAP activities are integrated to form a bipolar spindle, we have developed a novel high-throughput, image-based combinatorial RNAi screening methodology in human cells. Using a custom siRNA library targeting MAPs we have performed an RNAi interaction screen using cells where we have also depleted ch-TOG by shRNA. Depletion of ch-TOG results in multipolar spindle formation^12^,^29^. By quantifying spindle phenotypes after MAP depletion in this background, we determine how spindle assembly pathways interact. Through statistical analysis and downstream validation of individual genes and gene clusters, we describe a network of genetic interactions that is responsible for coordinating the activity of spindle assembly pathways. We show that ch-TOG interacts with a diverse set of MAPs in order to coordinate bipolar spindle assembly. Since ch-TOG is a key regulator of spindle assembly, this represents a core network of interactions required for spindle formation in human cells. Through this network of interactions, we propose that ch-TOG promotes MT polymerisation from the centrosome that is not only robust to perturbation, but also ensures that spindle MT dynamic instability is maintained at a level that facilitates MT reorganisation into a bipolar structure and kinetochore attachment.

## Results

### A ch-TOG RNAi interaction screen identifies novel interactions between MAPs during mitotic spindle assembly

**Figure 1:**
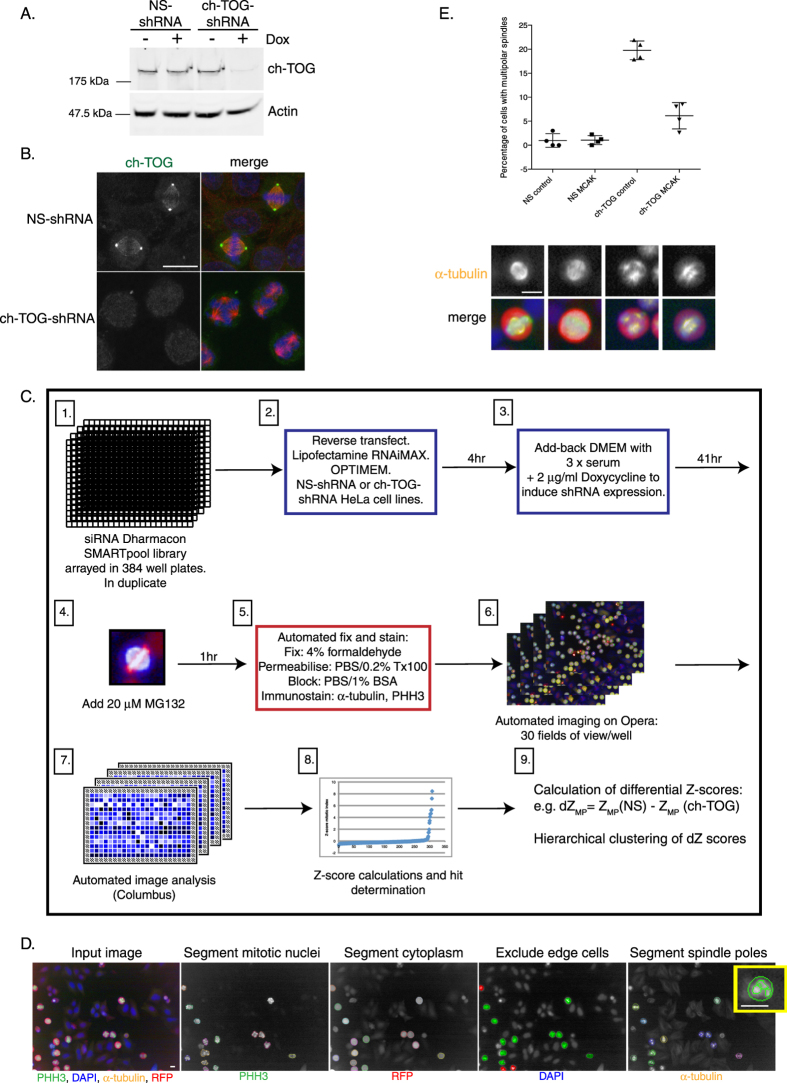
Setup of combinatorial RNAi screen. A. Western blot showing the extent of ch-TOG protein reduction 42 hr after Doxycycline (Dox) induction in a cell line stably expressing ch-TOG shRNA. β-actin is used as a loading control. B. Images showing that ch-TOG is depleted from the centrosomes and spindle MTs 42 hr after Dox addition. ch-TOG is in green, α-tub in red and DNA in blue in merged images. C. Workflow of RNAi screen. D. Image segmentation workflow to identify mitotic nuclei, segment mitotic cells and detect spindle poles. In yellow is a zoomed image from the final panel to show spindle pole segmentation. Scale bars are 10 μm. E. Graph showing the quantification of validation experiments. Mean +/− standard deviation (STD) of n =  4 is shown. Underneath the graph are representative images taken from the screening data to show the spindle phenotypes of each of the four conditions shown in the graph. α-tubulin is in yellow, PHH3 in green, RFP in red and DNA is in blue in merged images. All scale bars represent 10 μm.

To identify genes that interact with ch-TOG during spindle assembly, we first generated a stable, clonal HeLa cell line in which ch-TOG can be depleted in response to Doxycycline (dox). As a negative control, we integrated a non-silencing (NS) shRNA into HeLa cells (Methods). Upon addition of dox, ch-TOG protein is depleted in ch-TOG shRNA cells by approximately 90% and cells arrest in mitosis with multipolar spindles (Fig. 1A, B). Timelapse imaging of NS-shRNA and ch-TOG-shRNA cells expressing GFP-Tubulin 32 hr after dox addition confirmed that ch-TOG shRNA cells assemble stable multipolar spindles (Supplementary Movies S1,2), similar to ch-TOG knockdown by siRNA^29^,^30^.

When ch-TOG levels are reduced, multiple acentrosomal asters appear, particularly in the vicinity of chromatin upon nuclear envelope breakdown (NEBD; compare Supplemental Movies S1 and S2). Small acentrosomal MT asters then coalesce to form larger acentrosomal spindle poles that remain separated from the centrosomal asters, forming stable multipolar spindles (Supplemental Movie S2). Thus, consistent with previous reports, ch-TOG is necessary for the formation of a bipolar spindle^11^,^12^,^13^,^14^,^15^,^30^. Even in the absence of a separate centrosomal marker, it is possible to distinguish centrosomal from acentrosomal asters since they are present as two asters before NEBD and are larger and brighter than acentrosomal asters.

These observations, and previous reports^29^,^30^, suggest that depletion of ch-TOG compromises centrosome-mediated MT assembly. Furthermore, although other spindle assembly pathways are likely to be active in ch-TOG-deficient cells, we hypothesise that a bipolar spindle is not assembled because ch-TOG activity may also be necessary to integrate the activities of these additional pathways. This is in contrast to other MAPs that may regulate only MT dynamic instability and whose depletion does not result in the formation of multipolar spindles. In support of this hypothesis, ch-TOG and its homologues localise to MT-organising centres (MTOCs), at MT plus-ends, at kinetochores and along MT lattices, suggesting a diverse set of interactions and functions^51^. Together with observations that ch-TOG homologues promote MT plus-end dynamics *in vitro*^24^,^32^,^52^,^53^, these data suggest that the activity of ch-TOG is unlikely to be confined to simply promoting centrosomal MT polymerisation.

Therefore, to map the ch-TOG network in spindle assembly and to determine if we can identify genes that coordinate the action of different spindle assembly pathways in a ch-TOG dependent manner, we carried out an RNAi screen for spindle formation in unsensitised (NS-shRNA) and sensitised (ch-TOG-shRNA) cells using a custom-designed siRNA library targeting MAPs. Briefly, cells were reverse transfected with siRNA alone (NS background), or in the background of ch-TOG depletion (ch-TOG background; Fig. 1C). The screen was performed at a single timepoint in duplicate. We established an image analysis protocol to quantify spindle pole number and morphology using custom-made scripts (Methods). Mitotic cells were detected based on Ser10 Phospho-histone H3 staining (PHH3), segmented using RFP (coexpressed with the shRNA), and spindle poles detected using α-tubulin immunostaining (Fig. 1D). This protocol measures the percentage of cells with multipolar spindles, the average number of spindle poles per cell, the mitotic index, total cell number (a measure of viability), and the percentage of multinucleate cells. Screens were performed in duplicate and we obtained significant correlation of feature scores between plates (Fig S1A; p < 0.0001). Therefore, to calculate final Z-scores, normalised scores across duplicate plates were averaged (Methods; Fig S1B; Tables S1,2).

Depletion of ch-TOG by shRNA leads to an increased percentage of cells with multipolar spindles, an increase in spindle pole number per cell, and an increase in mitotic index (Fig S1A). Note that we measure the percentage of cells with multipolar spindles in ch-TOG depleted cells to be approximately 20% (19.67 +/−1.96; n = 4; Fig. 1E). This degree of multipolar spindles indicates that the depletion of ch-TOG is not complete, which will allow us to find both enhancers and suppressors of the ch-TOG phenotype. Codepletion of MCAK in our ch-TOG shRNA cells suppressed the multipolar spindle phenotype, confirming that our methodology can identify genuine genetic interactions (Fig. 1E; Table S2;^11^,^13^,^30^).

We analysed the data in two ways. First, a gene was considered to either suppress or enhance the ch-TOG multipolar spindle phenotype if gene depletion across replicates resulted in a mean percentage of cells with multipolar spindles more than +/− 1.5 standard deviations from the mean of control siRNA wells (Tables S1,2; Fig S1B). ch-TOG depletion from ch-TOG shRNA cells lead to a 1.8 fold increase in the percentage of cells with multipolar spindles, again indicating that ch-TOG depletion by shRNA is not complete. From 240 genes in the library, 3.3% of genes were classified as a hit in the NS-shRNA background (0 suppressors, 8 enhancers), rising to 11.7% in the ch-TOG shRNA background (13 suppressors, 15 enhancers). Notably, a number of genes known to have roles in bipolar spindle assembly in mammalian cells only scored as hits in the ch-TOG sensitised background, for example, the microtubule depolymerase MCAK, involved in MT-kinetochore attachment^54^, and the minus-end directed kinesin HSET^55^. This shows the importance of using combinatorial screens to determine the full repertoire of genes required for a given process under different conditions.

**Figure 2:**
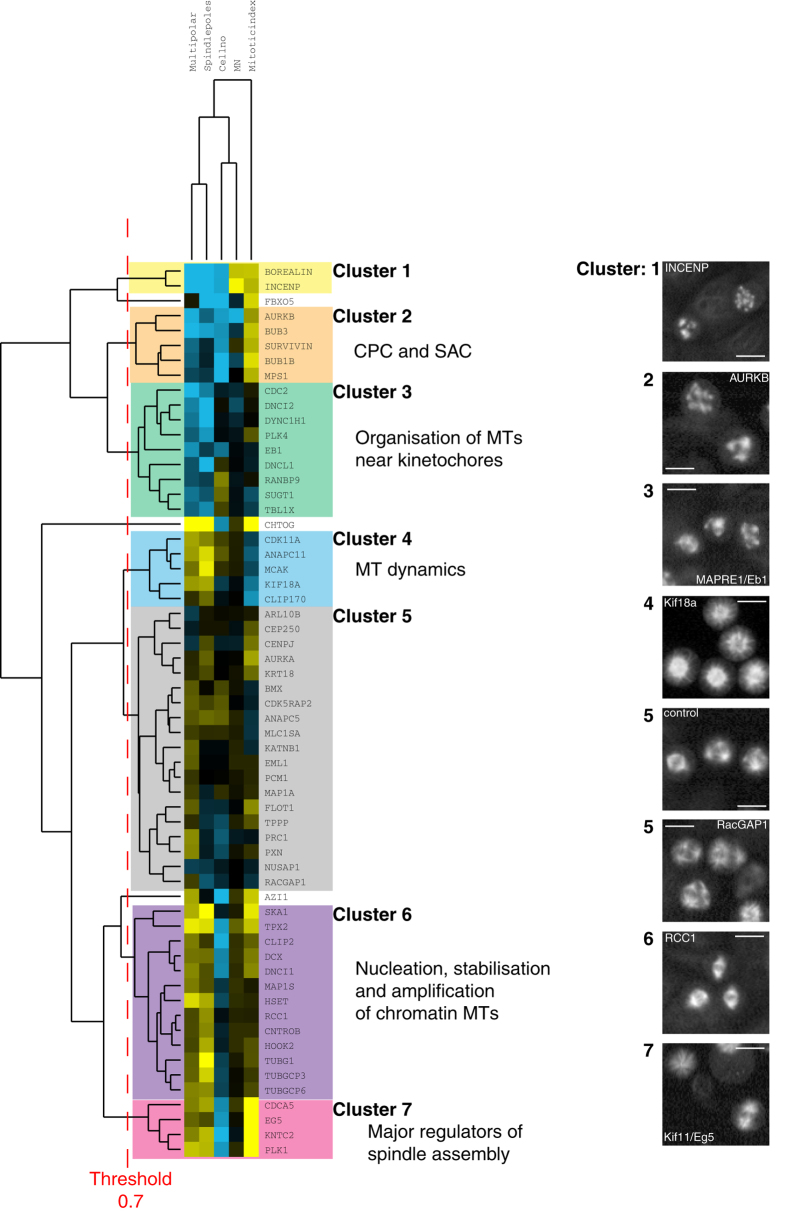
Hierarchical clustering of differential Z (dZ) scores. Only genes that had a Z-score > +/−1 for changes in either percentage of cells with multipolar spindles or number of spindle poles per cell in ch-TOG shRNA cells are included here. Yellow represents a positive dZ score, blue represents a negative dZ score and darker shading represents a dZ score close to zero. On the right are representative images of α-tubulin staining from the screen for some of the genes listed. ‘MN’ refers to percentage of multinucleate cells. All scale bars represent 10 μm.

We also calculated a differential Z-score (dZ;^56^,^57^) where the Z-score of the ch-TOG shRNA background is subtracted from that of the NS-shRNA background for each gene (Methods, Table S3). The dZ score provides a measure of how the phenotype of a particular gene depletion changes between the NS- and ch-TOG-shRNA backgrounds. The larger the dZ score, the larger the difference in phenotype. We used hierarchical clustering by complete linkage of dZ scores across five features (percentage of cells with multipolar spindles, number of spindle poles per cell, cell number, mitotic index and percentage of multinucleate cells) to identify clusters of genes with similar phenotypes in ch-TOG depleted cells (Fig. 2, Fig S2). Genes are considered to be part of phenotypically distinct groups when they cluster together above a cluster distance metric (an average of uncentred Pearson correlation coefficients) greater than 0.7 (see Methods). This generated seven clusters (Fig. 2), with grouped genes showing similarity in known functions and interactions with MAPs.

Cluster 2 (Fig. 2, orange) contains genes whose depletion increases the percentage of ch-TOG shRNA cells with multipolar spindles, the average number of spindle poles per cell and cell number. Depletion of these genes also slightly increases the percentage of multinucleate cells and decreases mitotic index. This cluster contains the Chromosome Passenger Complex (CPC) proteins Aurora B and Survivin plus the Spindle Assembly Checkpoint (SAC) proteins Bub3, Bub1B and Mps1. This suggests that the failure to engage CPC or SAC signalling in ch-TOG deficient cells leads to further defects in spindle assembly as these cells are allowed to progress through to mitotic exit even in the presence of unattached kinetochores. Cluster 3 (Fig. 2, green) contains genes whose depletion also increases the percentage of ch-TOG shRNA cells with multipolar spindles and the number of spindle poles per cell, but unlike genes included in Cluster 2, their depletion has little effect on cell number or mitotic index. Three components of the Cytoplasmic dynein complex (DNCI2, DYNC1H1 and DNCL1) are members of this cluster. Cluster 3 also contains the MT plus-tip tracking protein, EB1 and two mitotic kinases: CDC2 and PLK4. Cluster 6 (Fig. 2, purple) contains genes whose depletion decreases the percentage of ch-TOG shRNA cells with multipolar spindles, the number of spindle poles per cell, the mitotic index and the percentage of multinucleate cells but slightly increases cell number. This cluster includes a smaller subcluster of three genes: TUBG1, TUBGCP3 and TUBGCP6 that physically interact as part of the gamma-tubulin ring complex (γ-TuRC;^58^,^59^). Cluster 6 also contains proteins implicated in MT crosslinking and/or stabilisation (HSET, MAP1S, CENTROBIN, DCX) and proteins required for chromatin-mediated MT nucleation (TPX2, RCC1). Cluster 4 (Fig. 2, blue) contains genes whose depletion decreases the percentage of ch-TOG shRNA cells with multipolar spindles, the number of spindle poles per cell, increases the mitotic index and has little effect on the cell number or the percentage of multinucleate cells. This cluster contains genes known to regulate MT growth and stability, particularly at the plus end of MTs (MCAK, KIF18A, CLIP170). Together, these data show that clustering of dZ scores effectively groups genes known to physically or functionally interact (e.g. members of the γ-TuRC, components of the Dynein complex), or that localise to the same subcellular location (e.g. CPC components), during spindle assembly.

In some cases, gene depletion results in phenotypes in both unsensitised and sensitised backgrounds. Cluster 5 (Fig. 2, grey) contains a large group of genes with low dZ scores, i.e. these genes have similar phenotypes in NS and ch-TOG shRNA backgrounds (Fig. 2). This cluster includes, for example, genes essential for cytokinesis (PRC1 and RACGAP1) that increase the percentage of multipolar spindles in both NS and ch-TOG backgrounds, likely due to failures in the final stages of cell division. Cluster 7 (Fig. 2, pink) is also present in both NS and ch-TOG shRNA cells (Fig. 2, S2) and includes proteins essential for centrosome separation (EG5 and PLK1;^60^,^61^), chromatin cohesion (CDCA5/SORORIN;^62^,^63^) and MT-kinetochore attachment (KNTC2/HEC1;^64^). Depletion of genes in this cluster induces severe mitotic defects in both NS and ch-TOG shRNA backgrounds, reflecting their major, upstream roles in spindle assembly and/or mitotic progression. Although depletion of genes in Cluster 7 leads to spindle phenotypes in both NS and ch-TOG shRNA backgrounds, unlike genes in Cluster 5, their depletion generates a quantitatively different phenotype in each background. For example, depletion of genes in Cluster 7 decreases the percentage of ch-TOG shRNA cells with multipolar spindles, the number of spindle poles per cell and the mitotic index; and increases cell number (Fig. 2). Therefore, these genes appear to genetically interact with ch-TOG during spindle assembly.

Based on these analyses, we propose that a network of ch-TOG interactions simultaneously regulates MT nucleation, MT stability, MT growth and motor function to generate a bipolar spindle.

### ch-TOG reduces the contribution of chromatin-mediated microtubule nucleation to the initial stages of spindle assembly

**Figure 3:**
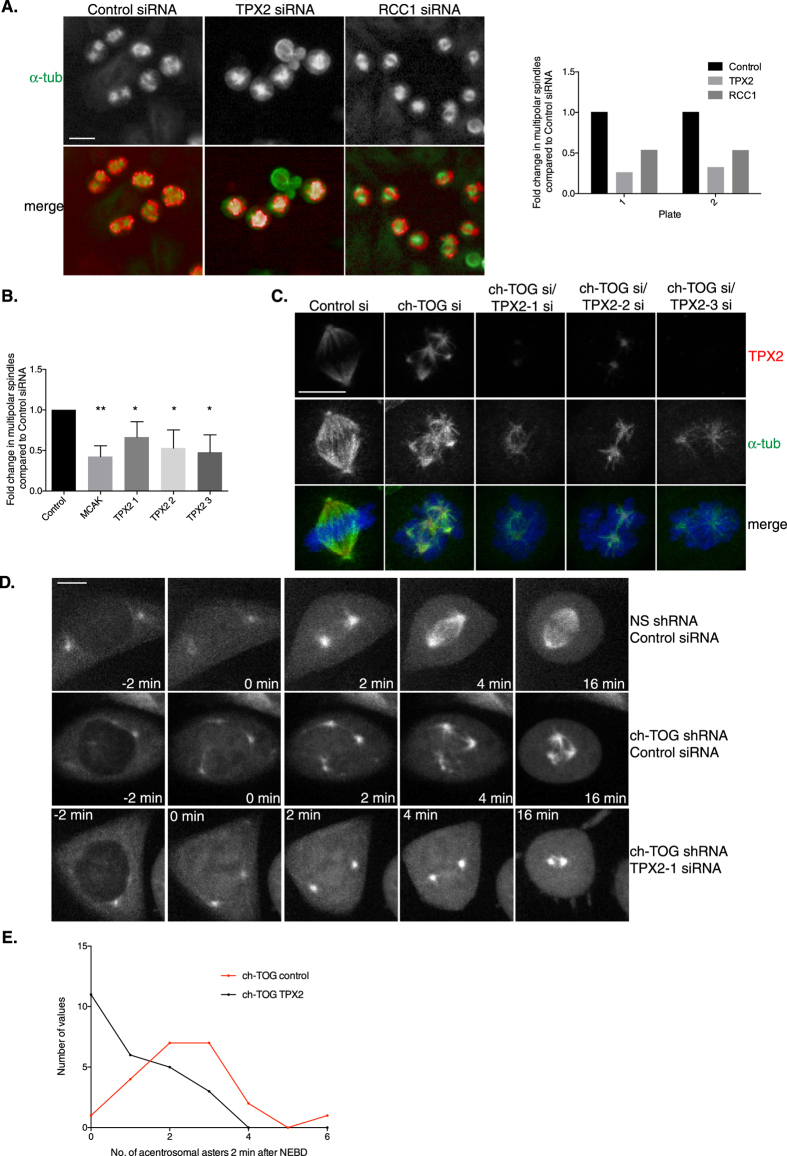
Suppressing the chromatin-mediated spindle assembly pathway restores bipolar spindles to ch-TOG shRNA cells. A. Representative images of screening data of control, TPX2 and RCC1 siRNA in ch-TOG shRNA cells. α-tubulin is shown in green and PHH3 in red in merged images. Scale bar represents 10 μm. Graph shows the fold change in the percentage of cells with multipolar spindles across duplicate plates in the screen. B. Graph showing the validation of screening data with three independent siRNAs targeting TPX2. MCAK siRNA was included as a positive control. n = 3, mean +/− STD are shown. One-way ANOVA followed by Dunnett’s test for multiple comparisons: **p ≤ 0.001, *p ≤ 0.05. C. Images from synchronised cells showing depletion of TPX2 using three siRNAs and loss of multipolar spindle formation in ch-TOG+TPX2 codepleted cells. TPX2 is in red, α-tubulin in green and DAPI in blue in merged images. Scale bar represents 10 μm. D. Stills taken from live imaging of GFP-Tubulin NS and ch-TOG shRNA cells treated with control or TPX2-1 siRNA. All images are aligned to NEBD at 0 min. Images shown are maximum intensity projections of Z-stacks. Scale bar represents 5 μm. E. Graph showing the frequency distribution of acentrosomal aster appearance in ch-TOG shRNA cells treated with either control or TPX2-1 siRNA. Acentrosomal asters were scored 2 min post-NEBD, n = 22 cells (control) and n = 25 cells (TPX2). Student’s t-test determined the difference between the two distributions to be significant (p = 0.0002).

We first sought to understand the relationship between ch-TOG and members of Cluster 6 involved in chromatin-mediated spindle assembly. RCC1 and TPX2 are two genes in Cluster 6 required for chromatin-mediated spindle assembly in many cell types^65^,^66^,^67^,^68^. Deficiency of RCC1 or TPX2 restores the ability of ch-TOG deficient cells to form a bipolar spindle structure (Figs. 2, 3A). We note that this bipolar structure is likely not functional since the MT mass in these spindles is considerably attenuated compared to wild-type spindles. We have validated our screening data with three independent siRNAs targeting TPX2 (Fig. 3B). Depletion of TPX2 in ch-TOG shRNA cells also leads to cell death after 42 hr. To eliminate this effect we used a synchronisation protocol to observe the effects of TPX2 and ch-TOG depletion in cells during their first mitosis after protein depletion by RNAi (Methods). Both TPX2 and ch-TOG protein levels were efficiently reduced using this protocol (Fig. 3C, S3A). Moreover, in synchronised ch-TOG+TPX2 co-depleted cells we still observe suppression of multipolar spindle assembly (Fig. 3C). Based on these data we hypothesise that inhibition of chromatin-mediated spindle assembly in ch-TOG deficient cells prevents the formation of multipolar spindles because the formation of acentrosomal spindle structures is severely compromised by the inhibition of chromatin-mediated MT polymerisation, which would otherwise compensate for the loss of centrosome-mediated spindle assembly.

To determine if suppression of multipolar spindle formation after TPX2 depletion in ch-TOG shRNA cells was mediated through a reduction in chromatin-mediated MT assembly, we examined spindle assembly in real-time after depletion of ch-TOG and TPX2. In NS-shRNA cells only centrosomal nucleated MTs are visible (Fig. 3D, Movies S1,2). We never observed MT nucleation around chromatin in the presence of ch-TOG protein. We hypothesise that this is because in wild-type cells, MT polymerisation at centrosomes occurs earlier and faster than chromatin-mediated MT polymerisation, and thus outcompetes chromatin-mediated pathways for tubulin and/or makes chromatin-mediated assembly difficult to image. In contrast, in ch-TOG shRNA cells, MT nucleation around chromatin is clearly observed following NEBD. Depletion of TPX2 in ch-TOG shRNA cells severely reduces the appearance of acentrosomal MT asters at NEBD, indicating a reduction in chromatin-mediated MT nucleation (Fig. 3D, S3B; Movie S3). As a result, very short bipolar spindles form in ch-TOG+TPX2 co-depleted cells (25/25 cells observed formed bipolar spindles). From 25 ch-TOG+TPX2 codepleted cells, 11/25 (44%) have no acentrosomal asters shortly after NEBD (Fig. 3E). In the remaining 14 cells, acentrosomal asters form but are fewer in number and smaller in size than in ch-TOG cells and are rapidly incorporated into a bipolar spindle (Fig. 3E). Based on these data we propose that ch-TOG promotes robust centrosomal MT nucleation that overwhelms or outcompetes the potential assembly of acentrosomal spindle structures that is driven by chromatin-mediated MT polymerisation. However, chromatin-mediated MT polymerisation is still needed for the generation of spindle MTs in wild-type cells, and ch-TOG activity is also required to ensure acentrosomal spindle MTs are incorporated into the centrosomal spindle.

### A role for the γ-tubulin ring complex in maintaining acentrosomal spindles

**Figure 4:**
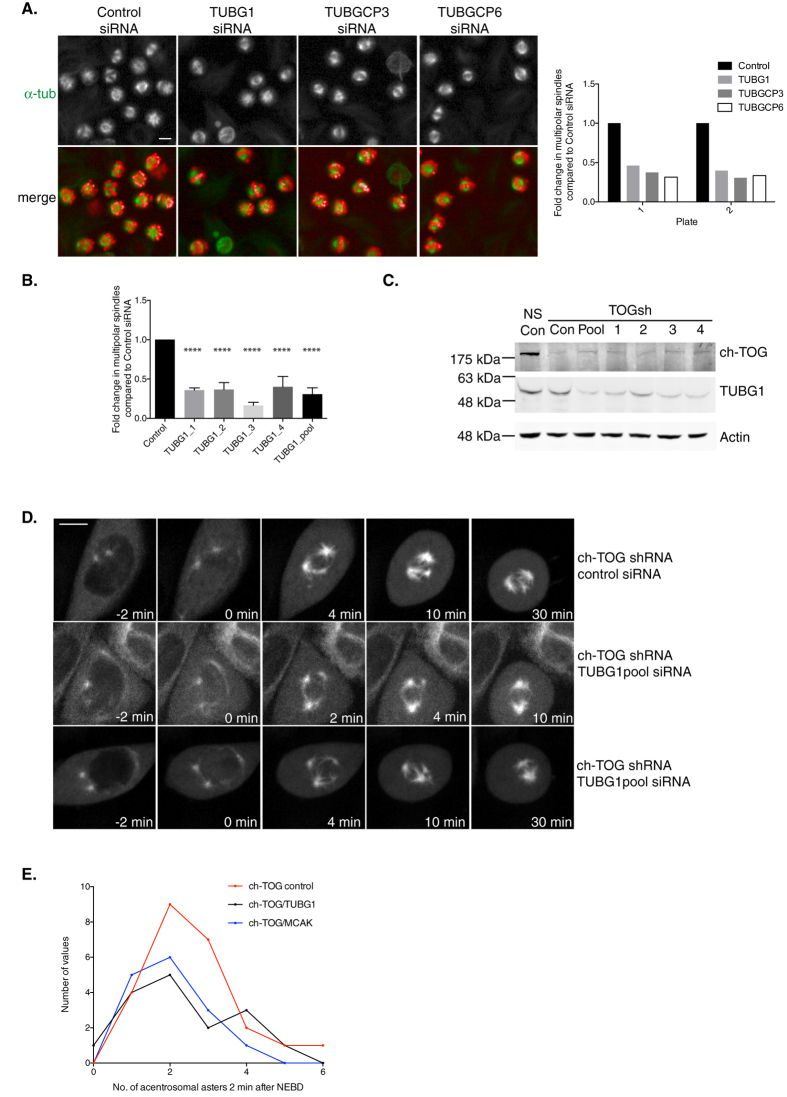
Depleting γ-TuRC components restores bipolar spindle formation to ch-TOG depleted cells. A. Representative images of screening data of control, TUBG1, TUBGCP3 and TUBGCP6 siRNA in ch-TOG shRNA cells. α-tubulin is shown in green and PHH3 is shown in red in merged images. Scale bar represents 10 μm. Graph shows the fold change in the percentage of cells with multipolar spindles across duplicate plates in the screen. B. Graph showing the validation of our screening data with four independent siRNAs targeting TUBG1 and a re-test of the TUBG1 pooled siRNA. n = 3, mean +/− STD are shown. One-way ANOVA followed by Dunnett’s test for multiple comparisons: ****p ≤ 0.0001. C. Western blot showing decrease in ch-TOG protein in ch-TOG shRNA cells and γ-tubulin protein after TUBG1 siRNA. Β-actin is used as a loading control. D. Stills taken from movies of ch-TOG shRNA cells expressing GFP-Tubulin treated with either control siRNA or TUBG1pool siRNA. All images are aligned to NEBD at 0 min. Images shown are maximum intensity projections of Z-stacks. Scale bar represents 10 μm. E. Graph shows the frequency distribution of acentrosomal aster appearance in ch-TOG shRNA cells treated with either control, TUBG1pool or MCAK siRNA. Acentrosomal asters were scored 2 min post-NEBD, n = 24 cells (control), n = 16 cells (TUBG1), n = 15 cells (MCAK).

The γ-TuRC catalyses MT nucleation and is enriched at centrosomes and along spindle MTs^69^. Whilst depletion of four γ-TuRC components targeted by siRNA in our MAP library had no effect on the percentage of cells with multipolar spindles in NS-shRNA cells, depletion of 3/4 components of the γ-TuRC restored bipolar spindle formation in ch-TOG shRNA cells: TUBG1, TUBGCP3 and TUBGCP6 (Fig. 4A; depletion of TUBGCP5 had no phenotype). TUBG1, TUBGCP3 and TUBGCP6 depletion also exhibited similar dZ scores across all features, forming a subcluster within Cluster 6 (Fig. 2). We confirmed our screening data with four independent siRNAs targeting the core complex component, TUBG1 (Fig. 4B,C).

We hypothesised that the γ-TuRC may play a role in the nucleation of chromatin-mediated MTs, similar to that of TPX2. To test this, we used live imaging of spindle assembly in cells depleted of ch-TOG and TUBG1 (Fig. 4D). To our surprise, the number of acentrosomal asters forming around chromatin after NEBD in ch-TOG+TUBG1 codepleted cells was similar to that in ch-TOG+control siRNA depleted cells (Fig. 4D,E; p = 0.72, one-way ANOVA). This demonstrated that, unlike TPX2, the γ-TuRC is not required for acentrosomal spindle assembly. However, we observed that these acentrosomal asters were rapidly incorporated into centrosomal asters to form a bipolar spindle in ch-TOG+TUBG1 codepleted cells (Fig. 4D, Movies S4,5). This is similar to the phenotype observed after ch-TOG+MCAK codepletion (Fig. 4E, ch-TOG+control vs ch-TOG+MCAK p = 0.26, one-way ANOVA).

Through live imaging of NS+TUBG1 depleted cells, we observed that, as in wild-type cells, there was no MT nucleation in the vicinity of chromatin, and centrosomal MT nucleation still appeared to be the dominant source of spindle MTs post NEBD (Movie S6). Also, centrosomal MT nucleation did not appear to be reduced in ch-TOG+TUBG1 codepleted cells compared to ch-TOG+control siRNA depleted cells (Fig. 4D). Since we do not observe a reduction in centrosomal or chromatin-mediated MT assembly after depletion of TUBG1 from ch-TOG depleted cells, our data are more consistent with a model where γ-TuRC acts after the initial steps of MT aster nucleation around chromatin and centrosomes, possibly to amplify MT assembly by promoting branching nucleation^70^,^71^. Therefore we propose that in the presence of ch-TOG, γ-TuRC promotes branching MT nucleation and these MTs are incorporated into the spindle structure by ch-TOG driven activity. We hypothesise that the absence of γ-TuRC in ch-TOG deficient cells restores bipolar spindle assembly because in acentrosomal asters, the chromatin pathway may be insufficient to maintain MT mass and the γ-TuRC may be required to amplify MTs further. In centrosomal asters, there may be a reduced requirement for branching MT nucleation because centrosomes and chromatin both contribute to maintaining MT polymer mass. Taken together, we propose that γ-TuRC is necessary for the maintenance of the MT mass polymerised by chromatin.

### ch-TOG limits the amount of HSET binding to spindle microtubules

**Figure 5:**
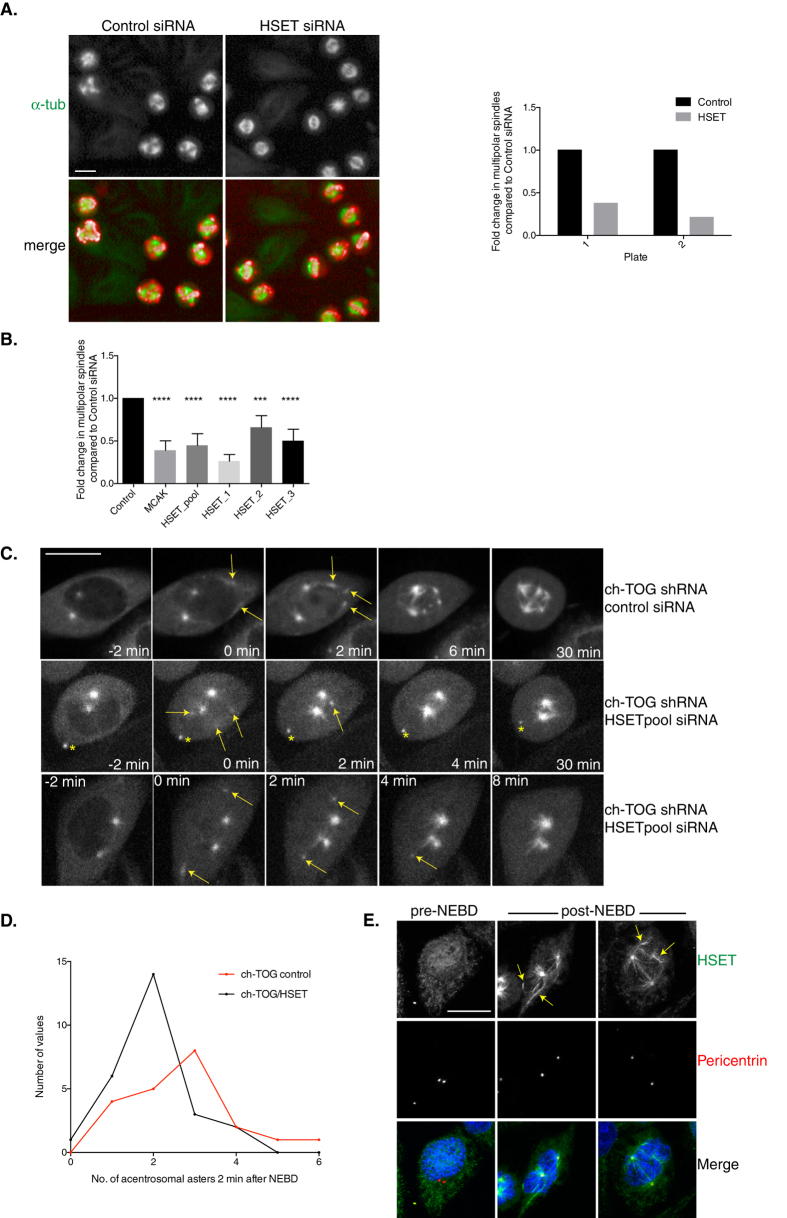
ch-TOG and HSET interact during mitotic spindle formation. A. Representative images of control and HSET depleted ch-TOG shRNA cells. α-tubulin is in green and PHH3 is in red in merged images. Graph shows the fold change in the percentage of cells with multipolar spindles across duplicate plates in the screen. B. Graph showing the validation of our screening data with three independent siRNAs targeting HSET and a re-test of the HSET pooled siRNA. MCAK siRNA was included as a positive control in all experiments. n = 3, mean +/− STD is shown. One-way ANOVA followed by Dunnett’s test for multiple comparisons: ****p ≤ 0.0001, ***p ≤ 0.001. C. Stills taken from movies of ch-TOG shRNA cells expressing GFP-Tubulin treated with either control siRNA or HSETpool siRNA. All images are aligned to NEBD at 0 min. Yellow arrows indicate acentrosomal asters, which are smaller in ch-TOG+HSET codepleted cells. Yellow asterisk indicates non-specific fluorescence (outside the cell at −2 min). Images shown are maximum intensity projections of Z-stacks. Scale bar represents 10 μm. D. Graph shows the frequency distribution of acentrosomal aster appearance in ch-TOG shRNA cells treated with either control or HSETpool siRNA. Acentrosomal asters were scored 2 min post-NEBD, n = 21 cells (control), n = 26 cells (HSET). Student’s t-test determined the difference between the two distributions to be significant (p = 0.025). E. Representative images of ch-TOG shRNA cells in the process of undergoing NEBD. Prior to NEBD, HSET is nuclear. As the nuclear envelope breaks down, HSET is released and localises to acentrosomal MT asters and bundles (indicated by yellow arrows), as well as centrosomal MTs. HSET is green, Pericentrin is red and DNA is blue in merged images. All scale bars represent 10 μm.

Our screen showed that depletion of HSET in ch-TOG shRNA cells restored bipolarity to spindles (Fig. 2; Tables S1, 2). HSET is a minus-end directed kinesin, a member of the kinesin-14 family. Kinesin-14 motors cross-link and slide MTs polewards, focussing MT minus ends. In the absence of centrosomes, kinesin-14 members are essential to form spindle poles yet, in the presence of centrosomes, are redundant for spindle pole formation^55^,^72^. We validated our screening data with three independent siRNAs (Fig 5B, S4A). These data show that the depletion of HSET can suppress multipolar spindle formation in ch-TOG depleted cells. This is surprising given that depletion of components of a second microtubule minus-end directed motor, cytoplasmic dynein, gives the opposite phenotype (enhances multipolar spindle formation in ch-TOG shRNA cells) and recent data have shown that HSET is required to cluster acentrosomal and centrosomal poles in some cancer cell lines^73^.

To explore how a reduction in HSET could restore bipolarity to ch-TOG depleted spindles, we performed live cell imaging in GFP-tubulin expressing ch-TOG shRNA cells treated with either control or HSET siRNA (Fig 5C). In ch-TOG+HSET codepleted cells, acentrosomal asters appear upon NEBD but are smaller in size and fewer in number (Fig 5 C,D; Supplemental Movies S7,8). Acentrosomal asters move towards the centrosomal poles and coalesce with centrosomal MT asters to form a bipolar spindle. The moderate reduction in the number of acentrosomal asters in cells where ch-TOG and HSET have been co-depleted could reflect an inability to nucleate, or focus and stabilise, MT asters in the absence of HSET activity. Consistent with a role for HSET in the stabilisation of acentrosomal asters in ch-TOG depleted cells, we observe HSET localising to acentrosomal MT asters and bundles immediately upon NEBD (Fig 5E). We also observe that depletion of ch-TOG leads to an increase in the amount of HSET localising to the mitotic spindle (Fig S4B,C). Importantly, an increase in spindle-bound HSET was not due to an increase in acentrosomal MTs since we still observed an increase in HSET in bipolar mitotic spindles formed after ch-TOG and MCAK codepletion (Fig S4D). Also, total cellular levels of HSET were not increased in ch-TOG depleted mitotic cells (Fig S4E). Increased HSET levels on the spindle after ch-TOG depletion may be due to either a reduction in MT dynamics or due to competition with ch-TOG for MT binding. To test the former possibility, we treated HeLa cells with low doses of nocodazole (NZ) to suppress MT dynamics. We found a dose-dependent increase between the concentration of NZ and the amount of HSET localised to the spindle (Fig S4F). This indicates that either HSET binds more stably to less dynamic MTs or that less dynamic MTs recruit more HSET. Taken together, our data imply that ch-TOG maintains a dynamic MT population, in part, by limiting the binding of the MT cross-linking protein, HSET. These data are also consistent with the presence of additional proteins in the same cluster as HSET that have MT-stabilising properties, such as MAP1S, CENTROBIN and DCX^74^,^75^,^76^,^77^ (Fig. 2; Tables S1,2). Furthermore, these data identify an important role for HSET in stabilising acentrosomal asters in human cells when MT dynamics are compromised.

### The balance between Dynein and Eg5 activity during bipolar spindle assembly is altered in the absence of ch-TOG

**Figure 6:**
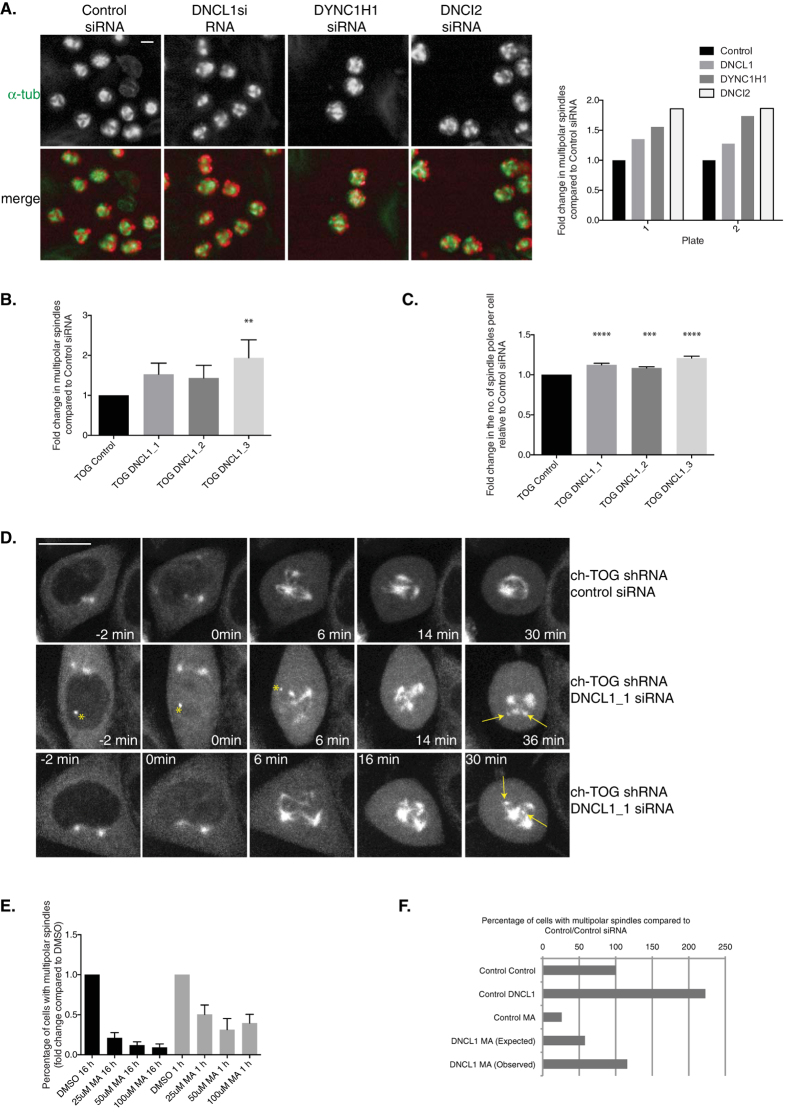
ch-TOG maintains a balance between microtubule motor activities. A. Representative images of screening data of control, DNCL1, DYNC1H1 and DNCI2 siRNA in ch-TOG shRNA cells. α-tubulin is in green and PHH3 is in red in merged images. Scale bar represents 10 μm. Graph shows the fold change in the percentage of cells with multipolar spindles across duplicate plates in the screen. B. Graph showing the validation of our screening data with three independent siRNAs targeting DNCL1. n = 3, mean +/− STD is shown. One-way ANOVA followed by Dunnett’s test for multiple comparisons: **p ≤ 0.01. C. Graph showing an increase in the number of spindle poles in ch-TOG shRNA cells after depletion of DNCL1. n = 3, mean +/− STD is shown. One-way ANOVA followed by Dunnett’s test for multiple comparisons: ****p ≤ 0.0001, ***p ≤ 0.001. D. Stills taken from movies of ch-TOG shRNA cells expressing GFP-Tubulin treated with either control siRNA or DNCL1_1 siRNA. All images are aligned to NEBD at 0 min. Yellow arrows indicate small acentrosomal asters in DNCL1 siRNA cells that are still present at late timepoints. Yellow asterisk indicates non-specific fluorescence. Images shown are maximum intensity projections of Z-stacks. Scale bar represents 10 μm. E. Graph showing the percentage of cells with multipolar spindles in ch-TOG shRNA cells treated with either DMSO or MA, added either pre spindle assembly for 16h or post spindle assembly, for 1h. All data is normalised to DMSO treated cells. n = 2, mean +/− STD is shown. F. Graph showing an interaction between Eg5 and DNCL1 in ch-TOG shRNA cells (DNCL1_3 siRNA was used in this experiment). To determine if an interaction occurs, an interaction score was calculated based on the difference between the observed and expected phenotypes (see Methods). We determined a positive/alleviating genetic interaction score of +1.02.

Cluster 3 contains three components of the cytoplasmic dynein complex: DNCL1, DYNC1H1 and DNCI2. Dynein is a large minus-end directed motor that can crosslink and slide antiparallel MTs to focus MT minus ends^78^,^79^. Depletion of any of these three components leads to an increase in the percentage of cells with multipolar spindles in ch-TOG shRNA cells (Fig. 2, 6A, Tables S1, 2). We validated our screening data with three independent siRNAs targeting DNCL1 (Fig 6B, S5A, B). From the three DNCL1 siRNAs we tested, all three showed an increase in the percentage of cells with multipolar spindles but only one of these (DNCL1_3) was statistically significant. However, we observed that these ch-TOG shRNA cells depleted of DNCL1 appeared to have more spindle poles per cell than ch-TOG shRNA cells treated with control siRNA. Dynein is required to drive the coalescence of small acentrosomal MT asters and bundles with centrosomal asters, to form a bipolar spindle^80^,^81^ and immunofluorescence data has shown that the dynein adaptor, p150, is present at ch-TOG acentrosomal poles^11^. Therefore, we wondered if in ch-TOG depleted cells, dynein was driving the coalescence of small acentrosomal asters to form larger, stable acentrosomal asters. We analysed the number of spindle poles per cell after depletion of DNCL1 in ch-TOG shRNA cells and found a statistically significant increase in the number of spindle poles per cell with all three DNCL1 siRNAs, indicating a defect in aster coalescence in these cells (Fig 6C). Together, these data show that dynein normally acts to limit the extent of multipolar spindle formation in ch-TOG depleted cells.

We used live imaging of GFP-Tubulin to determine if a reduction in DNCL1 in ch-TOG shRNA cells does cause an increase in the number of spindle poles per cell through a defect in aster coalescence. In ch-TOG shRNA cells treated with control siRNA, asters coalesce and a stable multipolar spindle is formed (Fig 6D). However, in ch-TOG shRNA cells treated with DNCL1 siRNA we observe a reduction in aster coalescence, such that small MT asters persist, and cells do not achieve a stable multipolar spindle with spindle poles appearing to move more than in ch-TOG shRNA only depleted cells (Fig 6D, Movies S9,10). Together, these data suggest that ch-TOG may promote dynein activity during spindle assembly to drive aster coalescence into a bipolar spindle.

Whilst it appears that dynein can promote the coalescence of small acentrosomal asters, it is unable to promote the final coalescence of acentrosomal poles with centrosomal poles in ch-TOG depleted cells. This is likely due to the antagonistic activity of Eg5. Eg5 is a plus-end directed kinesin that can crosslink and slide apart antiparallel MTs^82^,^83^,^84^. Eg5 generates antagonistic forces to dynein during spindle assembly^85^,^86^. Consistent with previous reports, our screen shows that depletion of Eg5 from ch-TOG depleted cells reduces the percentage of cells with multipolar spindles (Fig. 2, Tables S1,2;^11^,^87^). We confirmed our screening data using the Eg5 inhibitor, Monastrol (MA; Fig 6E). These data also show that Eg5 is required to maintain multipolar spindles in ch-TOG depleted cells since if MA was added during the last hour of spindle assembly (i.e. once a multipolar spindle had formed) the percentage of cells with multipolar spindles was reduced (Fig 6E). These data suggest that ch-TOG acts to maintain a balance between the opposing motor activities of dynein and Eg5 during spindle assembly.

To confirm an interaction between dynein and Eg5 in ch-TOG depleted cells, we depleted DNCL1 and inhibited Eg5 in ch-TOG shRNA cells. As predicted, we observed an epistatic interaction between dynein and Eg5 activity (Fig 6F). These data confirm that ch-TOG acts to maintain a balance between Eg5 and dynein motor activities to promote bipolar spindle assembly.

## Discussion

**Figure 7:**
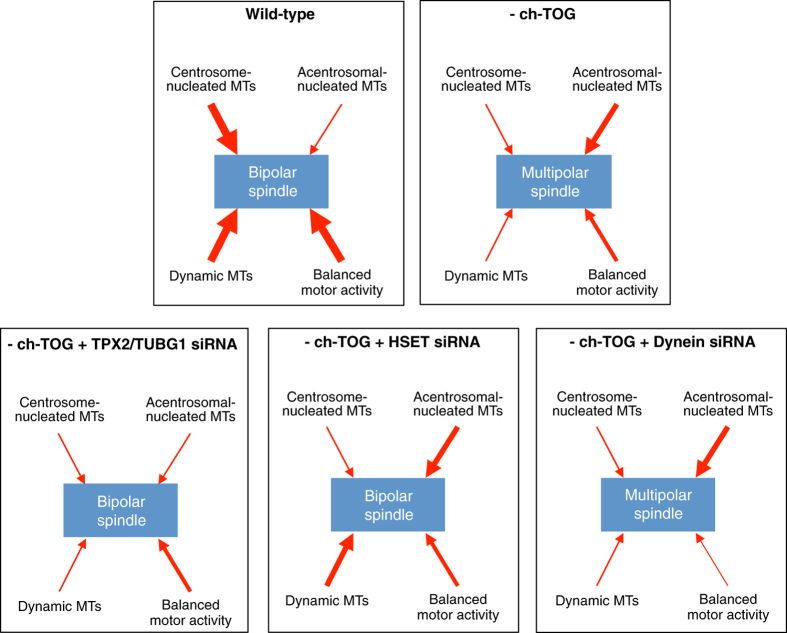
A network of ch-TOG interactions ensures bipolar spindle formation. In wild-type cells, MTs nucleated from the centrosome are dominant in spindle formation. Depletion of ch-TOG not only reduces the contribution of centrosomal MTs to the spindle, but also reduces MT dynamics and leads to an imbalance in motor activity. Depletion of TPX2 or TUBG1 in ch-TOG depleted cells reduces the contribution of acentrosomal MTs to spindle formation, thus redressing the balance between centrosomal and acentrosomal pathways. Depletion of HSET in ch-TOG depleted cells reduces MT crosslinking and increases overall MT dynamics allowing acentrosomal asters to be incorporated into centrosomal asters. Depletion of dynein in ch-TOG depleted cells leads to an increased imbalance in motor activity and generates additional spindle poles.

Promoting robust MT assembly from two centrosomes provides an intuitive mechanism to ensure spindle bipolarity. However, the existence of additional spindle assembly pathways suggests that in human somatic cells, centrosome-mediated spindle assembly may not be sufficient to generate a robust mechanism for spindle assembly and segregation of chromosomes^35^,^66^. The existence of multiple spindle assembly pathways may also represent a mechanism by which the spindle structure can explore diverse configurations in search of those that are functional. We present evidence here that a network of proteins around ch-TOG integrates the actions of multiple MT assembly pathways to promote bipolar spindle assembly. We propose that ch-TOG mediated integration is a key means by which spindle assembly can achieve robustness to perturbation whilst retaining a capacity for structural exploration (Fig. 7).

In particular, our data supports a model whereby ch-TOG enables spindle bipolarity by both maintaining a dominant centrosomal spindle assembly pathway and also integrating MTs nucleated by additional pathways into the centrosomal array. Specifically, whilst we and others have shown that in the absence of ch-TOG, centrosomal MT nucleation is reduced^29^,^30^, using live imaging we demonstrate that depletion of ch-TOG also leads to an increase in the contribution of acentrosomal pathways to the initial stages of spindle formation. In our live imaging assays, we only monitored GFP-Tubulin and did not include a centrosomal marker. Based on these data we cannot formally rule out that acentrosomal asters are not being nucleated by centrosomal fragments. However, we do not believe this is the case for three reasons: 1. depletion of TPX2, a protein essential for chromatin-mediated spindle assembly, severely reduces the generation of acentrosomal asters (Fig. 3D,E); 2. examination of cells immediately post-NEBD shows that acentrosomal asters are not associated with the PCM component, Pericentrin (Fig 5E); 3. we have shown in previous work that centrosome fragmentation is not a major contributor to multipolar spindle formation in ch-TOG depleted cells^30^. The data shown here from human cells are consistent with recent observations in *Drosophila* embryos showing an increase in alternative spindle assembly pathways in the absence of a robust centrosomal pathway^6^. Thus in the absence of ch-TOG, while MTs polymerised by chromatin do contribute to overall MT mass, these MTs are not incorporated into the bipolar array. We show that by reducing the activity of the chromatin-mediated (TPX2, RCC1) and potentially intra-spindle mediated ( γ-TuRC, TPX2^88^) assembly pathways in ch-TOG depleted cells, we can restore bipolar spindle formation because chromatin-mediated polymerisation of acentrosomal MTs is compromised. These data suggest that chromatin-mediated MT assembly not only allows the generation of MT mass to be robust to perturbations that affect centrosomal MT polymerisation, but also allows MTs to explore configurations beyond those that are normally restricted by MT nucleation from two centrosomes. ch-TOG activity is required to ensure that this robustness and conformational flexibility are balanced.

We show that by promoting MT dynamics, ch-TOG may limit the over-stabilisation of MT asters by MT-crosslinking proteins, such as HSET, MAP1S, CENTROBIN and DCX (cluster 5, Fig. 2; Tables S1,2;^74^,^75^,^76^,^77^). Consistent with this, we show that depletion of HSET in ch-TOG shRNA cells can restore bipolar spindle formation. The ability of ch-TOG to maintain long, dynamic MTs^30^,^51^ may explain why cells depleted of ch-TOG form multipolar spindles and not bipolar spindles as formed after, for example, centrosome ablation^2^ since a reduction in MT dynamics after ch-TOG depletion prevents acentrosomal asters from being organised into a bipolar spindle. ch-TOG is able to maintain long dynamic MTs through i) its inherent MT polymerase activity^52^, ii) synergistic interactions with MT plus-end binding proteins, such as EB1 (Fig. 2; Tables S1,2;^28^,^32^,^89^, and iii) counterbalancing the activity of MT depolymerising proteins, such as MCAK and KIF18A (cluster 3, Fig. 2; Tables S1,S2;^29^,^90^,^91^). Finally, by maintaining a population of dynamic MTs, ch-TOG activity ensures that the balance between the opposing motor activities of EG5 and dynein is maintained, which is required for bipolar spindle assembly^87^.

Together, our data suggest that if ch-TOG is depleted from cells, the following events occur (Fig. 7). When centrosome-mediated polymerisation is compromised, chromatin-mediated MT assembly plays a more dominant role in the early stages of spindle assembly, leading to the formation of acentrosomal asters (Fig. 3). In the absence of ch-TOG, the unchecked MT depolymerising activities of MCAK and KIF18A (Fig. 2) prevent the growth of long MT arrays and reduce the overall spindle MT polymer mass. Branching MT nucleation, stimulated by γ-TuRC, can occur from pre-existing MTs to increase/maintain MT mass throughout the spindle, in both centrosomal and acentrosomal asters (Fig. 4). The reduction in MT dynamics results in acentrosomal asters becoming stabilised, which prevents the incorporation of acentrosomal MTs into centrosomally-nucleated asters. The inability to incorporate acentrosomal MTs into the spindle structure is aggravated by the increased binding of proteins that can crosslink MTs, such as HSET, MAP1S, DCX and CENTROBIN (Fig 5). A reduction in the formation of long dynamic MTs also prevents dynein-driven coalescence of acentrosomal asters with centrosomal asters and instead favours the EG5-mediated separation of MT asters (Fig 6), leading to the stabilisation of a multipolar spindle configuration.

ch-TOG depletion in HeLa and the K562 leukaemia cell lines causes multipolar spindle formation^13^. However, in hTert-RPE1 cells, ch-TOG depletion does not lead to multipolar spindle formation (our unpublished data). Our results show that the phenotype observed after ch-TOG depletion is likely to depend on the concentration of other MAPs present in the cell. For example, a cell line that usually expresses only low levels of HSET may not form multipolar spindles after ch-TOG depletion. This may reflect differences between cell lines originating from different tissues or differences between transformed and non-transformed cell lines. This is a significant question for future research since anti-cancer drugs targeting spindle assembly would be predicted to have different efficacies, and side effects, in different tissues. For example, the response to Taxol has been shown to be dependent on cellular levels of the MAPs Ensconsin^38^ and Tau^39^.

In this screen, we have focussed on developing and validating a robust screening platform to find redundant, synergistic and antagonistic MAP activities required for ch-TOG driven spindle assembly. This screen was performed at a single timepoint in duplicate, however, we have shown that this strategy is successful in revealing novel interactions required for spindle assembly and has furthered our understanding of how the key spindle assembly protein, ch-TOG, acts to build a bipolar spindle. The goal of this paper was not to characterise in great mechanistic detail how the selected proteins interact with ch-TOG during spindle assembly, but to demonstrate how combinatorial screens of this type can be used to reveal novel interactions required for spindle assembly and that the interactions identified can be validated with independent reagents. Here we have identified a ch-TOG-centric network of genetic interactions required for bipolar spindle assembly. The goal now is to carry out additional combinatorial screens using other key spindle assembly regulators as sensitising backgrounds to build up a genetic interaction network of spindle assembly in human cells. Furthermore, by clustering genes based on their phenotypes across several features, we can uncover novel spindle assembly regulators and, by comparison to the phenotypes of known proteins, we can begin to assign potential functions to uncharacterised proteins.

## Methods

### Cell culture

HeLa cells were maintained in DMEM + 10% FBS (Sigma) + 1% Penicillin/Streptomycin (P/S; Invitrogen) and kept at 37 °C and 5% CO2. NS-shRNA and TOG-shRNA HeLa cell lines were maintained in complete media plus 1 μg/ml Puromycin (Thermo Fisher).

### Generating NS-shRNA and ch-TOG shRNA cell lines

The pTripZ Doxycycline-inducible shRNA system from Thermo Scientific was used to deplete ch-TOG in HeLa cells. Doxycycline addition induces the expression of an mRNA coding for the shRNA and RFP, separated by an IRES sequence. Therefore cells expressing shRNA can be selected based on RFP expression. pTripZ shRNA vectors targeting ch-TOG were purchased from Thermo Scientific. Five shRNA sequences were tested and clone A11 was deemed to give the best depletion of ch-TOG in HeLa cells. A non-silencing (NS) shRNA was generously provided by Pascal Meier (Breakthrough Research Centre, Institute of Cancer Research, London, UK).

Lentiviral transduction of HeLa cells was carried out according to the manufacturer’s protocol (Thermo Scientific). After Lentiviral infection, cells were selected in 1 μg/ml Puromycin for 10–14 days. Cells were then expanded and sorted into 96-well plates to single-cell density. Single cell clones were allowed to expand and 24 individual clones were picked. Clones were split into a 384 well PerkinElmer Cell Carrier plate in duplicate. To half the wells, 2 μg/ml Doxycycline was added to induce shRNA expression. 48 hr later, cells were fixed, immunostained and imaged (see below). Those clones with the highest RFP expression and highest number of cells with multipolar spindles were selected. Western blotting and immunostaining with anti-ch-TOG confirmed that these clones had reduced ch-TOG protein.

### siRNA library

The MAP siRNA library was a generous gift of Spiros Linardopoulos (Institute of Cancer Research, London, UK). Each gene in the library is targeted by a Dharmacon OnTargetPlus (OTP) pool of four siRNAs. The library was diluted to a 10 μM stock and arrayed onto black 384 well PerkinElmer Cell Carrier plates using the Echo Liquid Handler (Labcyte). The final siRNA concentration per well is 40 nM. In addition, OTP pool siRNAs targeting MCAK (suppresses multipolar spindle formation in ch-TOG depleted cells) and RacGAP1 (induces cytokinesis failure) were added to the plate as positive controls.

### Reverse transfection

All screens were performed at a single timepoint in duplicate. A Multidrop Combi Reagent dispenser (Thermo Scientific) was used throughout the transfection protocol to ensure even liquid addition. 5 μl of OPTIMEM (Gibco) was added to each well to redissolve the siRNA. Plates were centrifuged and incubated at RT for 5 min. Lipofectamine RNAimax (Invitrogen) was added in OPTIMEM to a final concentration of 8 μl Lipofectamine to 1 ml OPTIMEM and incubated at RT for 5 min. 5 μl of OPTIMEM/Lipofectamine mix was then added to the plates. Plates were centrifuged and incubated at RT for 20 min. NS-shRNA and ch- TOG shRNA HeLa cells were trypsinised and counted using an automated cells counter (Countess; Invitrogen). Cells were centrifuged at 1000 xg for 4 min. Media was aspirated and cells were resuspended in OPTIMEM to a final concentration of 2 × 10^5^ cells per ml. 10 μl of cell suspension was added to the plates, plates were centrifuged and incubated at 37 0C for 4 hr. After 4 hr, 10 μl of DMEM + 30% FBS + 3% P/S and 6 μg/ml Doxycycline (Dox; Sigma) was added to the plates (final concentration 10% FBS, 1% P/S and 2  μg/ml Dox). Plates were centrifuged and incubated at 37 °C for 41 hr.

### Fixing and immunostaining of plates

Before fixation, 10 μl cell medium plus 80 μM of the proteasome inhibitor MG132 (Tocris) was added to all plates for 1 hr (final concentration of MG132 is 20 μM). All fixing and staining was carried out using the PerkinElmer Cell::Explorer system coupled to automated liquid handling equipment. Solutions were dispensed using a Multidrop Combi Reagent dispenser (Thermo Scientific) and aspirated/washed using a Biotek washer with 96 pins. Plates were fixed by adding an equal volume of pre-warmed (37 °C) 8% formaldehyde/PBS solution to the wells and incubated at 37 °C for 10 min. Cells were then permeabilised in pre-warmed PBS/0.2% Triton X-100 for 10 min at RT. Cells were then blocked in 1% BSA/PBS (blocking solution) for 1 hr at RT. Primary antibodies were diluted in blocking solution: MTs were labelled using an anti-α-tubulin antibody (1:1000, mouse, Cat no. A11126, Invitrogen) and mitotic cells were labelled using anti-Ser10 PhosphoHistone H3 (1:1000, rabbit, Cat no. 06–570, Millipore). Cells were incubated with primary antibodies for 2 hr at RT, then washed three times in PBS. Secondary antibodies were diluted in blocking buffer: anti-mouse Alexa 488 and anti-rabbit Alexa 647 (Invitrogen). Cells were incubated in secondary antibody for 1hr at RT, then washed three times in PBS. Cells were finally incubated in 1 μg/ml Hoescht (H 33258, Sigma) diluted in PBS for 15 min at RT before a final wash in PBS and imaging.

### Imaging of RNAi screen

All plates were imaged using the PerkinElmer Opera high-content confocal screening platform with spinning disc. 30 fields of view per well were images using a 20x air objective, NA 0.45. On average, this generated images of 30–40 NS-shRNA and 250–300 ch-TOG-shRNA mitotic cells per well.

### Automated Image analysis

All Image analysis was performed using custom algorithms built in the Columbus software (PerkinElmer). Image analysis protocols are summarised below.

### Multipolar spindle detection, Cell number, Mitotic index and Multinucleate Cell determination

All nuclei were detected and segmented using Hoechst staining (total cell number). Intensity of PHH3 was measured in each nucleus and mitotic nuclei were determined using a minimum PHH3 intensity threshold (mitotic index). The cytoplasm of mitotic nuclei was segmented using RFP expression. Cells touching the edge of the image (incomplete segmentation) and poorly segmented cells (cells below a roundness threshold of 0.8) were excluded at this point. Tubulin intensity was measured in each mitotic cell and cells with very low tubulin immunostaining (where individual spindle poles could not be distinguished, below a mean of 30) were excluded since these are poorly segmented in later steps. Spindle poles were detected using the Spot Finder building block. Briefly, this detects spindle poles based on local intensity maxima and we defined individual poles as having a relative spot intensity of greater than 0.095 and a splitting coefficient of 0.56. This successfully defined individual spindle poles, and data shown in Fig 1E was validated by manually scoring randomly selected fields. Cells with greater than two spindle poles were classed as multipolar. To detect multinucleate cells, nuclei and mitotic nuclei were detected as above but this time, mitotic nuclei were excluded from the analysis. The cytoplasm of interphase cells was then segmented based on RFP localisation and the number of nuclei per cell scored to determine the percentage of cells with multipolar spindles.

### *HSET* spindle intensity measurements

Cells were stained with HSET and Pericentrin antibodies plus Hoechst for nuclei identification. All nuclei were identified and segmented based on Hoechst staining. Mitotic nuclei were identified as those cells that had a Hoechst intensity above a defined threshold. Mitotic spindles were segmented using HSET immunostaining and HSET intensity was measured within that area.

### Hierarchical Clustering

For clustering, only genes that had a standard deviation greater than +/− 1 from the mean for either the percentage of multipolar spindles per well or the number of spindle poles per cell in ch-TOG shRNA cells were analysed. Differential Z-scores (dZ scores; 56,57) were calculated by subtracting the Z-score of each feature for each gene for ch-TOG shRNA cells from that of the Z-score from NS-shRNA cells. Hierarchical clustering was used to cluster the resulting dZ scores using city-block distance and complete linkage in Cluster 3.0. Clustering data was visualised in TreeView. The cutoff for clusters was empirically set at 0.7 and was assigned based on genes with common functions clustering together (for example DNCI2, DYNC1H1 and DNCL1 in Cluster 3, Bub3 and Bub1B in Cluster 2, MCAK and Kif18A in Cluster 4).

### Correlation between replica plates

Correlation coefficients between replica plates were calculated by Spearman’s rank correlation test.

### RNAi

The following siRNAs (Dharmacon) were transfected into HeLa cells using Lipofectamine RNAiMAX (Invitrogen), all siRNAs used were ON-TARGETplus, unless otherwise stated: control siRNA 2, MCAK SMARTpool, HSET SMARTpool, ch-TOG SMARTpool, HSET 1 (5´-GCG GAA GUC CAG CAC UAU U-3´), HSET 2 (5´-GCA CCG GAA GUG GAA UUA A-3´), HSET 3 (5´-GGA CUC AAA CGU UGG ACC A-3´), TUBG1 SMARTpool, TUBG1-1 (5´AAAGAUCCAUGAGGACAUU), TUBG1-2 (5´-GAACCUGUCGCCAGUAUGA), TUBG1-3 (5´-CUAGAGGGCUUUGUGCUGU), TUBG1-4 (5´-UCUUAGAACGGCUGAAUGA), TPX2-1 (5´-GGACGAACCGGUAGUGAUA), TPX2-2 (5´-GCAUAAGGCAAAUCCAAUA), TPX2-3 (5´-GUACCAUUGUUAAGCCUUU), DNCL1_1 (5´-GUUCAAAUCUGGUUAAAAG), DNCL1_2 (5´-GAAGGACAUUGCGGCUCAU), DNCL1_3 (5´-GUACUAGUUUGUCGUGGUU).

### Live imaging of spindle assembly

NS- and ch-TOG-shRNA cells expressing GFP-Tubulin were imaged on the Opera HCS imaging system (PerkinElmer) fitted with an Environmental Control Chamber, using a 40xW objective. Cells were filmed at 37 °C and 5% CO_2_. Cells to be imaged were maintained in phenol-red free DMEM with 10% FBS and 1% P/S. Images were acquired as Z-stacks, every 1 μm and captured every 2 min. Images were then exported to Volocity (PerkinElmer) to visualise timelapse sequences. All movies are shown as extended focus projections.

### Western Blotting

Whole cell extracts were made by lysing HeLa cells directly in boiling sodium dodecyl sulphate (SDS) sample buffer. Extracts were separated by SDS-polyacrylamide gel electrophoresis using precast 4–20% Precise protein gels (ThermoScientific). Separated proteins were transferred to PVDF-FL at 80V for 90 min at 4 °C. PVDF membranes were blocked in 5% milk/TBS 10% glycerol for 1 hr at RT, incubated in primary antibodies overnight at 4 °C, washed 3 × 20 min in TBS/0.05% TritonX-100 (TBS/T) before incubating in Roti block (Roth) containing secondary antibodies at 1 μg/ml – either anti-mouse DyLight 680 or anti-rabbit DyLight 800 (Cell Signalling). Membranes were incubated in the dark for 1 hr at RT in secondary antibodies. Membranes were washed 3 × 20 min in TBS/T, 1x in water for 5 min before visualising on the LiCor imaging system. Protein levels were quantified using Odyssey software and all protein levels were normalised to B-actin in order to compare individual wells.

Primary antibodies used for Western blotting are anti-Tpx2 (Novus NB500-179; 1:1000), B-actin (Sigma, AC40; 1:5000), anti-Y-tubulin (Sigma, GTU-88; 1:2000), anti-HSET (Santa Cruz, M-63; 1:1000), anti-ch-TOG (QED Bioscience, 34032, 1:1000).

### Immunostaining cells

HeLa cells were plated onto coverslips in a 24-well plate. Cells were fixed in ice-cold Methanol at −20 °C for 5 min, washed in PBS/0.1% Tween (PBS/T) for 5 min at RT and blocked in 1% BSA/PBS/0.05% sodium azide for 1 hr at RT, or overnight at 4 °C. Primary antibodies were incubated in blocking buffer for 2 hr at 37 °C, coverslips were washed 3x in PBS/T before incubation with Alexa fluorophore-conjugated secondary antibodies (Invitrogen, 1:1000) in blocking buffer for 1 hr at 37 °C. Coverslips were washed 3x in PBS/T before Hoescht was added at 1 μg/ml in PBS for 15 min at RT. Coverslips were mounted on glass slides using Fluoromount-G (eBioscience). All coverslips were visualised on a Zeiss LSM710 confocal microscope and images captured using Zen software. All images were exported to Volocity software for analysis (PerkinElmer). Primary antibodies used for immunostaining are as above for Western blotting plus anti-α-tubulin (Sigma, DM1a; 1:2500), anti-centrin-3 (Abnova; 1:1000), anti-Eb1 (CRUK; 1:1000) and anti-Pericentrin (Abcam, ab4448; 1:1000).

### Cell synchronisation

HeLa cells were plated on coverslips and 2.5 mM thymidine added for 20 hr. Thymidine was removed by 3 washes in PBS and replaced with pre-warmed DMEM. After 5 hr, cells were washed 2x in PBS and placed in 0.4 ml OPTIMEM. siRNA was performed in OPTIMEM using lipofectamine RNAiMAX, according to the manufacturer’s instructions. After 4 hr, DMEM containing 3xFBS, 3xP/S and 7.5 mM Thymidine (2.5 mM final concentration) was added to transfected cells for 18 hr. After 18 hr, cells were washed 3x in PBS and placed in pre-warmed DMEM. 9–10 hr later, when cells could be seen to be entering mitosis cells were either fixed for immunostaining or processed for Western blotting.

### RT-PCR

Total RNA was extracted using the Rneasy plus mini kit, according to manufacturer’s instructions (Qiagen). 250 ng of RNA was converted to cDNA using random primers and MMLV reverse transcriptase, according to the manufacturer’s instructions (Invitrogen). Q-PCR was performed using SYBR Select Master Mix (Invitrogen) and analysed on a 7900HT Fast Real-time PCR system (Applied Biosystems). Relative mRNA levels were calculated using the delta delta Ct method. All data was normalised to GAPDH. Primers used to determine DNCL1 mRNA levels were DNCL1F: 5′-AGATGCAACAGGACTCGGTG, and DNCL1R: 5′-CCACTTGGCCCAGGTAGAAG.

### Interaction score

To determine if there was an interaction between DNCL1 and Eg5 in ch-TOG shRNA cells, we used the multiplicative method to detect genetic interactions and from this calculated an interaction score^92^. Briefly, this method calculates an expected phenotype from the product of the two single treatment phenotypes. The interaction score determines if the observed phenotype is significantly different to the expected phenotype.

### Drug treatments

Drugs used in this study are Eg5 inhibitor Monastrol (Tocris), Nocodazole (Sigma) and MG132 (Tocris). All drug compounds were diluted in DMSO and stored in aliquots at -20 °C.

## Additional Information

**How to cite this article**: Barr, A. R. and Bakal, C. A sensitised RNAi screen reveals a ch-TOG genetic interaction network required for spindle assembly. *Sci. Rep.* **5**, 10564; doi: 10.1038/srep10564 (2015).

## Supplementary Material

Supplementary Information

Supplementary Video S1

Supplementary Video S2

Supplementary Video S3

Supplementary Video S4

Supplementary Video S5

Supplementary Video S6

Supplementary Video S7

Supplementary Video S8

Supplementary Video S9

Supplementary Video S10

Supplementary Table S1

Supplementary Table S2

Supplementary Table S3
