# Supplementary material for: A sensitised RNAi screen reveals a ch-TOG genetic interaction network required for spindle assembly
Source: Sci Rep. 2015 Jun 3;5:10564. doi: 10.1038/srep10564 (PMC4453164; doi:10.1038/srep10564)
Supplement: Supplementary Information [file srep10564-s1.pdf]

A sensitised RNAi screen reveals a ch-TOG genetic interaction network required for spindle assembly

Alexis R. Barr and Chris Bakal

Supplemental Information

Supplemental Data

Supplementary Information

Supplementary Figure Legends

Figure S1. A Graphs to show the reproducibility between replicate plates for the five features measured across both backgrounds ($p<0.0001$). Only spindle pole number in NS-shRNA cells is not significant because well-to-well differences are very small. **B.** Z-score plots for the five features measured across both backgrounds. Green triangles represent control siRNA wells. Red squares are MCAK siRNA control wells.

Figure S2. Hierarchical clustering of Z-scores in the NS-shRNA background. Only genes that had a Z-score greater than +/- 1 for either the percentage of cells with multipolar spindles or the number of spindle poles per cell in the ch-TOG shRNA background are shown. Yellow indicates a positive Z-score (increase over control siRNA) and blue is a negative Z-score (decrease over control siRNA).

Figure S3. A. Western blot showing the depletion of TPX2 and ch-TOG after cell synchronisation (see Experimental Procedures). 1,2,3 labels above the wells represent TPX2-1,-2 and -3 siRNAs. B-Actin is used as a loading control. Graph shows the fold reduction in TPX2 levels (normalised to B-actin) relative to ch-TOG/control siRNA depletion. **B.** Stills taken from live imaging of GFP-Tubulin ch-TOG shRNA cells treated with TPX2-1 siRNA. Images are aligned to NEBD at 0 min. Images are maximum intensity projections of Z-stacks. Scale bar represents 5 μm.

Figure S4. A. Western blot showing ch-TOG and HSET depletion 42 hr after Dox addition. Β-actin was used as a loading control. Graphs on the right show the quantification of HSET and ch-TOG levels, normalised to Β-actin. **B.** Representative images of HSET staining during mitosis in NS-shRNA and ch-TOG shRNA cells. HSET is shown in green, α-tubulin in red, and DNA in blue in merged images. Scale bar represents 10 μm. **C.** Graph showing the quantification of HSET levels on spindles in mitotic cells. HSET levels are shown as median intensity fluorescence with interquartile range. Data shown are from one experiment but the same trends were observed across three independent experiments. No. of cells scored: NS/control: 180, NS/HSETsi: 310, ch-TOG/control: 1928, ch-TOG/HSETsi: 1766. ***$p<0.0001$ in student's t-test. **D.** Graph showing median intensity and interquartile ranges of spindle-bound HSET in ch-TOG/control cells and ch-TOG/MCAKsi cells. No. of cells scored: ch-TOG/control: 1928, ch-TOG/HSETsi: 2711. **E.** Western blot showing no overall increase in HSET levels in ch-TOG shRNA cells. Cells were transfected with control or HSETpool siRNA, then doxycycline added to induce NS or ch-TOG shRNA for 42 hours. For the last 16 hours, 40 ng/ml nocodazole was added to arrest all cells in mitosis. Β-actin is used as a loading control. **F.** Graph shows the relationship between nocodazole (NZ) concentration and spindle-bound HSET levels in mitotic cells. Median and interquartile range is shown. Data shown are from one experiment but the same trend was observed across three independent experiments

Figure S5. A. Graph showing fold reduction in DNCL1 mRNA after DNCL1 siRNA. All data was normalised to GAPDH. n=2. **B.** Western blot showing

the reduction of ch-TOG in ch-TOG/DNCL1 codepleted cells. Β-actin is used as a loading control. Graph shows quantification of ch-TOG levels, normalised to Β-actin.

Supplemental Tables

Table S1. Summary of Z-scores across five features for NS-shRNA cell line.

Table S2. Summary of Z-scores across five features for ch-TOG-shRNA cell line.

Table S3. Summary of dZ scores across five features.

Supplemental Movies

Movie S1. NS-shRNA expressing GFP-Tubulin and transfected with control siRNA.

Movie S2. ch-TOG-shRNA expressing GFP-Tubulin and transfected with control siRNA.

Movie S3. ch-TOG-shRNA expressing GFP-Tubulin and transfected with TPX2-1 siRNA.

Movie S4. ch-TOG-shRNA expressing GFP-Tubulin and transfected with TUBG1pool siRNA.

Movie S5. ch-TOG-shRNA expressing GFP-Tubulin and transfected with TUBG1pool siRNA.

Movie S6. NS-shRNA expressing GFP-Tubulin and transfected with TUBG1pool siRNA.

Movie S7. ch-TOG-shRNA expressing GFP-Tubulin and transfected with HSETpool siRNA.

Movie S8. ch-TOG-shRNA expressing GFP-Tubulin and transfected with HSETpool siRNA.

Movie S9. ch-TOG-shRNA expressing GFP-Tubulin and transfected with DNCL1_1 siRNA.

Movie S10. ch-TOG-shRNA expressing GFP-Tubulin and transfected with DNCL1_1 siRNA.

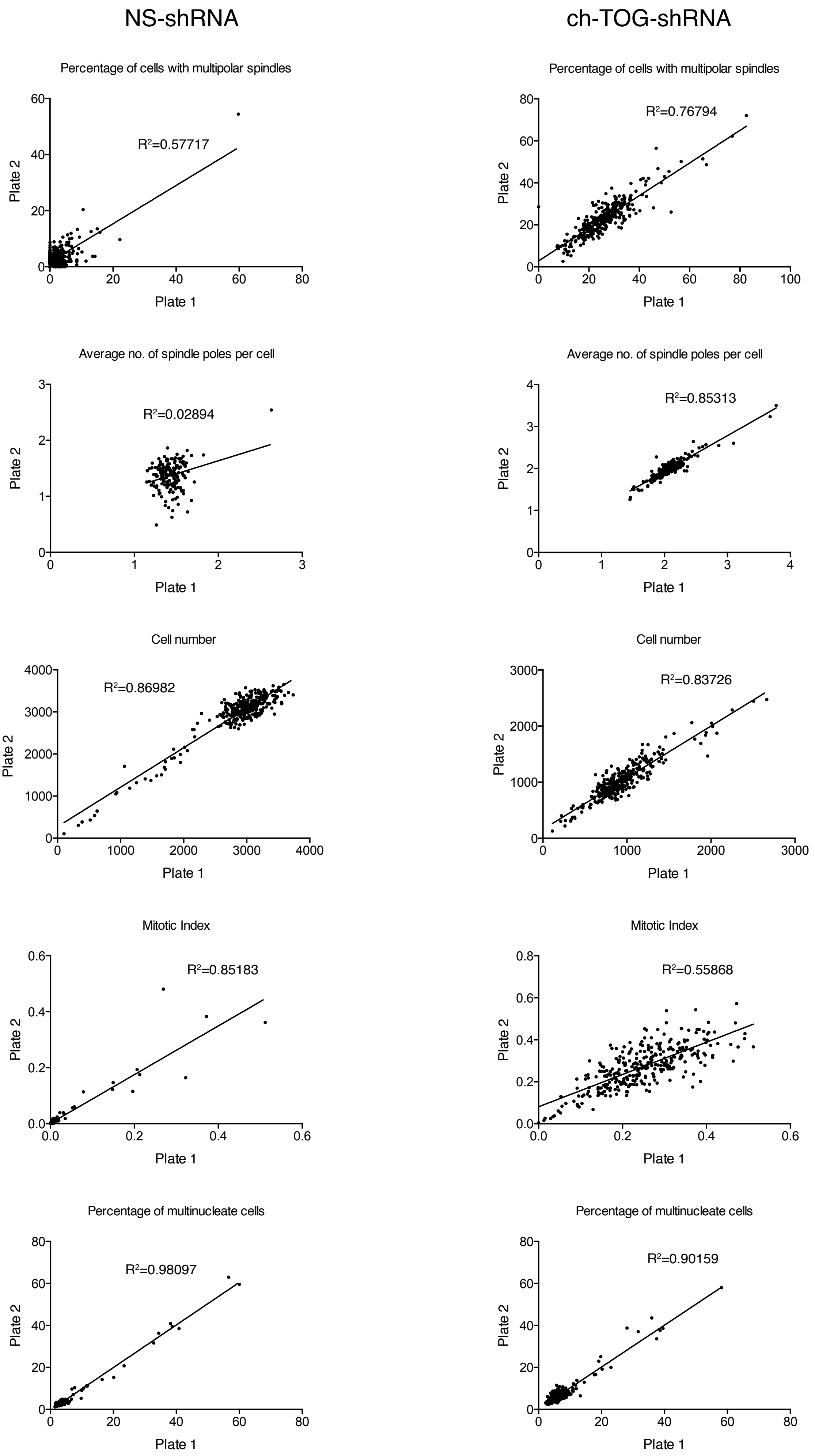
NS-shRNA
ch-TOG-shRNA
Percentage of cells with multipolar spindles
R²=0.57717
R²=0.76794
Average no. of spindle poles per cell
R²=0.02894
R²=0.85313
Cell number
R²=0.86982
R²=0.83726
Mitotic Index
R²=0.85183
R²=0.55868
Percentage of multinucleate cells
R²=0.98097
R²=0.90159
Plate 1
Plate 2

FIGURE S1A

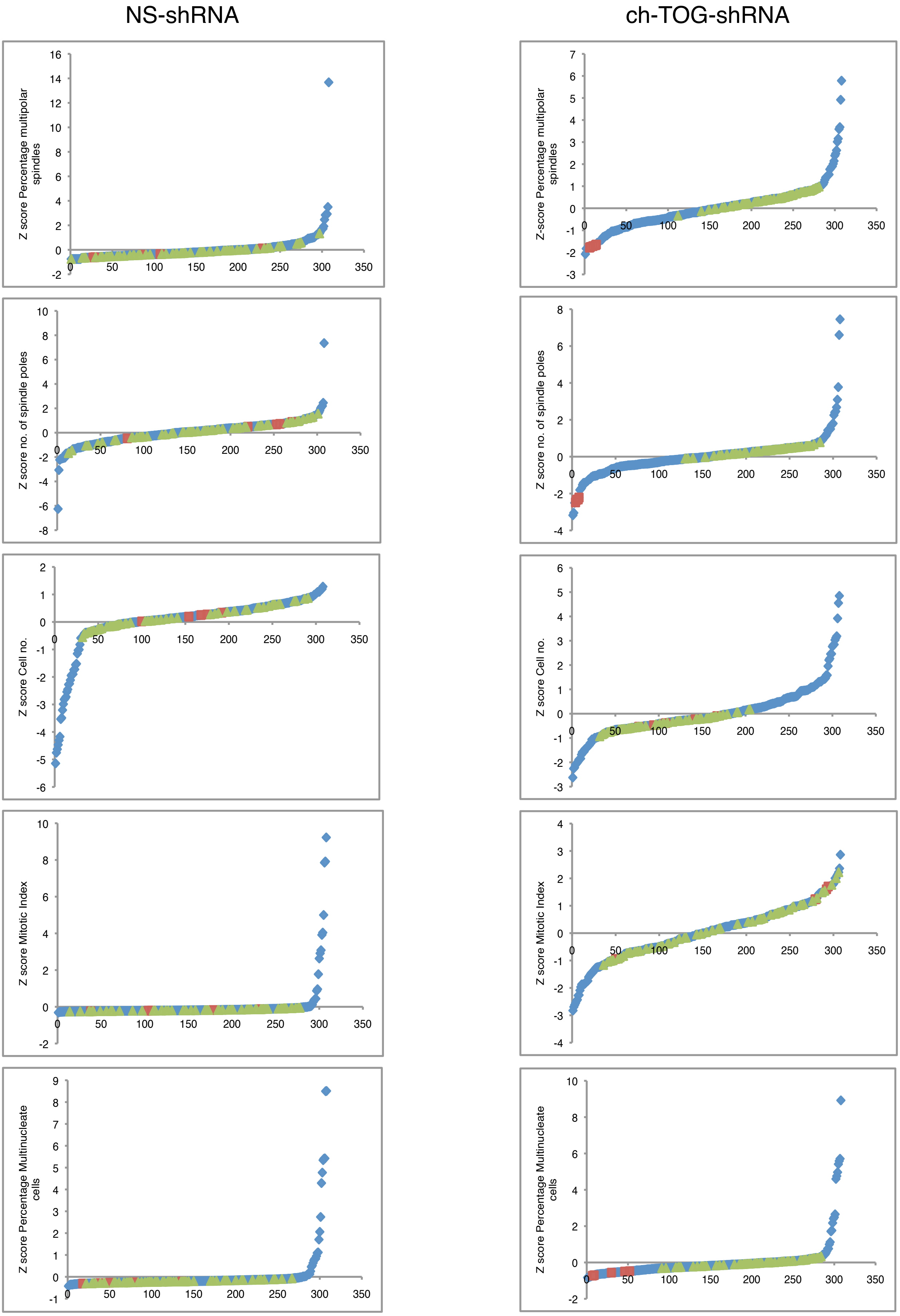
NS-shRNA
ch-TOG-shRNA
Z score Percentage multipolar spindles
Z-score Percentage multipolar spindles
Z score no. of spindle poles
Z score no. of spindle poles
Z score Cell no.
Z score Cell no.
Z score Mitotic Index
Z score Mitotic Index
Z score Percentage Multinucleate cells
Z score Percentage Multinucleate cells

FIGURE S1B

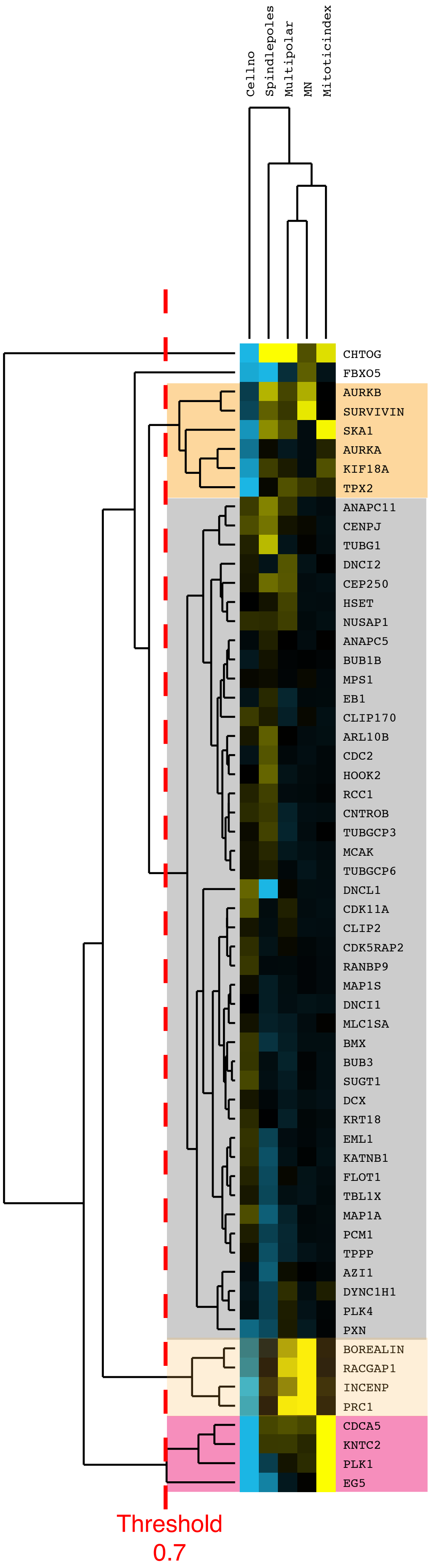

Cellno
Spindlepoles
Multipolar
MN
Mitoticindex
CHTOG
FBXO5
AURKB
SURVIVIN
SKA1
AURKA
KIF18A
TPX2
ANAPC11
CENPJ
TUBG1
DNCI2
CEP250
HSET
NUSAP1
ANAPC5
BUB1B
MPS1
EB1
CLIP170
ARL10B
CDC2
HOOK2
RCC1
CNTROB
TUBGCP3
MCAK
TUBGCP6
DNCL1
CDK11A
CLIP2
CDK5RAP2
RANBP9
MAP1S
DNCI1
MLC1SA
BMX
BUB3
SUGT1
DCX
KRT18
EML1
KATNB1
FLOT1
TBL1X
MAP1A
PCM1
TPPP
AZI1
DYNC1H1
PLK4
PXN
BOREALIN
RACGAP1
INCENP
PRC1
CDCA5
KNTC2
PLK1
EG5
Threshold
0.7

FIGURE S2

A.

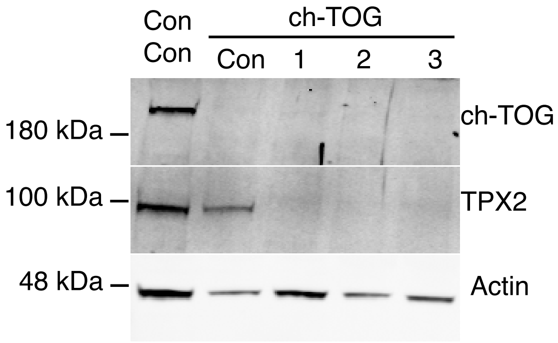
Con
Con
ch-TOG
Con
1
2
3
180 kDa
100 kDa
48 kDa
ch-TOG
TPX2
Actin

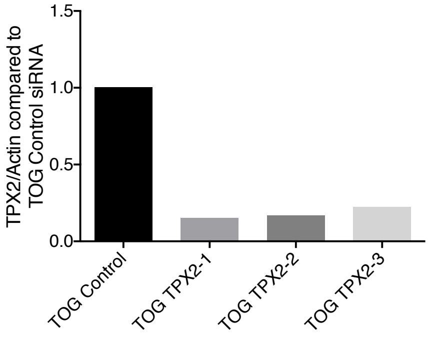
TPX2/Actin compared to TOG Control siRNA
1.5
1.0
0.5
0.0
TOG Control
TOG TPX2-1
TOG TPX2-2
TOG TPX2-3

B.

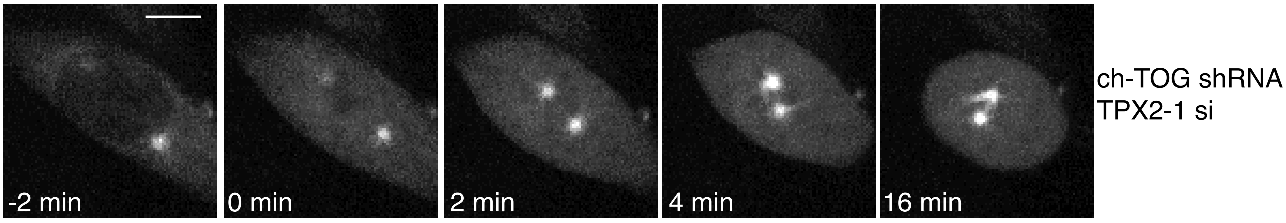
-2 min
0 min
2 min
4 min
16 min
ch-TOG shRNA
TPX2-1 si

FIGURE S3

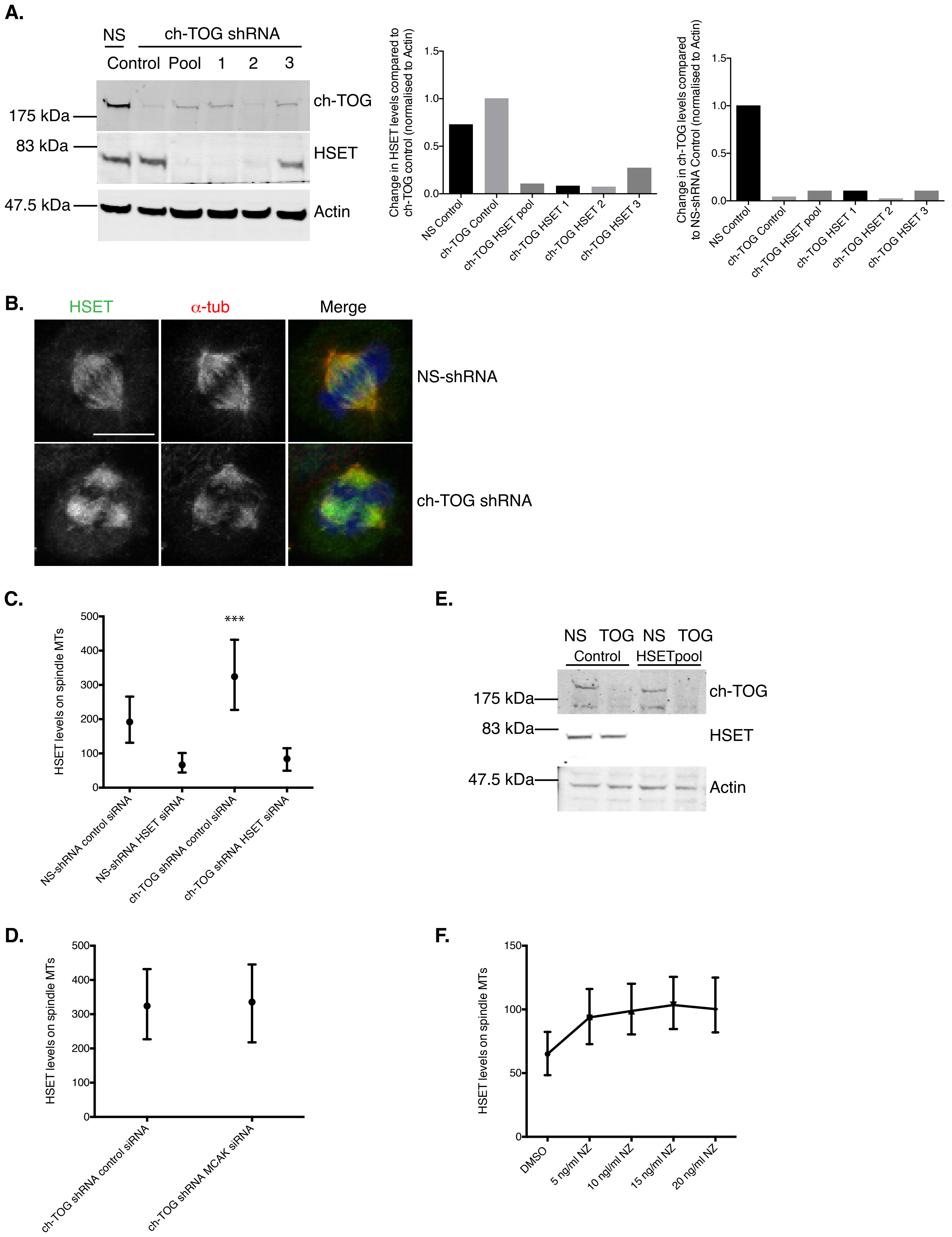
A.
NS
ch-TOG shRNA
Control
Pool
1
2
3
175 kDa
83 kDa
47.5 kDa
ch-TOG
HSET
Actin
Change in HSET levels compared to ch-TOG control (normalised to Actin)
1.5
1.0
0.5
0.0
NS Control
ch-TOG Control
ch-TOG HSET pool
ch-TOG HSET 1
ch-TOG HSET 2
ch-TOG HSET 3
Change in ch-TOG levels compared to NS-shRNA Control (normalised to Actin)
B.
HSET
α-tub
Merge
NS-shRNA
ch-TOG shRNA
C.
HSET levels on spindle MTs
500
400
300
200
100
0
***
NS-shRNA control siRNA
NS-shRNA HSET siRNA
ch-TOG shRNA control siRNA
ch-TOG shRNA HSET siRNA
D.
ch-TOG shRNA control siRNA
ch-TOG shRNA MCAK siRNA
E.
NS
TOG
Control
HSETpool
F.
150
50
DMSO
5 ng/ml NZ
10 ngl/ml NZ
15 ng/ml NZ
20 ng/ml NZ

FIGURE S4

A.

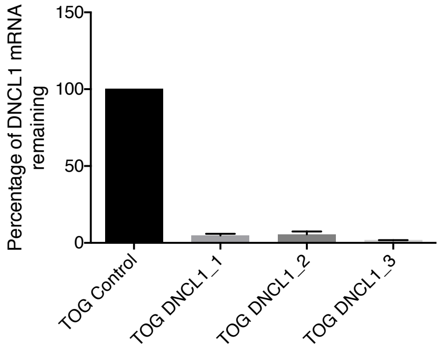
Percentage of DNCL1 mRNA remaining
150
100
50
0
TOG Control
TOG DNCL1_1
TOG DNCL1_2
TOG DNCL1_3

B.

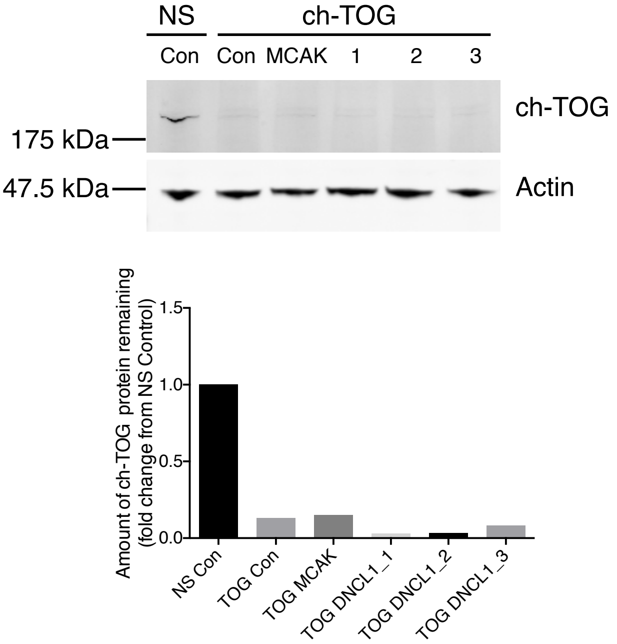
NS
ch-TOG
Con
Con
MCAK
1
2
3
ch-TOG
175 kDa
47.5 kDa
Actin
Amount of ch-TOG protein remaining (fold change from NS Control)
1.5
1.0
0.5
0.0
NS Con
TOG Con
TOG MCAK
TOG DNCL1_1
TOG DNCL1_2
TOG DNCL1_3

FIGURE S5
